# Kinetic Study of Depolymerization of Lactic and Glycolic Acid Oligomers in the Presence of Oxide Catalysts

**DOI:** 10.3390/polym12102395

**Published:** 2020-10-17

**Authors:** Vladimir Botvin, Svetlana Karaseva, Victor Khasanov, Anatoly Filimoshkin

**Affiliations:** 1 Department of High Molecular Compounds and Petrochemistry, Faculty of Chemistry, National Research Tomsk State University, 36 Lenin ave., 634050 Tomsk, Russia; svetlana_karasyova_1997@mail.ru (S.K.); filag05@rambler.ru (A.F.); 2 Department “New materials for Electrical and Chemical industries”, Faculty of Chemistry, National Research Tomsk State University, 36 Lenin ave., 634050 Tomsk, Russia; 3 Department of Organic Chemistry, Faculty of Chemistry, National Research Tomsk State University, 36 Lenin ave., 634050 Tomsk, Russia; serga01net@yandex.ru

**Keywords:** hydroxycarboxylic acid oligomers, depolymerization, metal oxides, cyclic diesters, isoconversional kinetics

## Abstract

For glycolic acid oligomers (GAO): l-lactic acid oligomers (LAO) mixtures, as an example, it was shown that the nature of the active sites of the catalysts significantly affect the depolymerization process. So, ZnO, which has mainly basic sites, leads to the highest yield of cyclic diesters. On the contrary, depolymerization in the presence of acidic γ-Al_2_O_3_ and without a catalyst is characterized by lower diester yields due to the prevalence of a side polycondensation reaction. Using GAO:LAO mixtures, it was shown that in the case of three studied systems (with ZnO, γ-Al_2_O_3_, and without catalyst), mixed interactions occurred, including *homo*-paired and *hetero*-paired intermolecular interactions, as well as intramolecular interactions of oligomeric molecules. Kinetic models of the processes under study were determined by isoconversional thermal analysis. In the case of depolymerization of oligomers in the presence of ZnO, the kinetic model was between the kinetic models of the first (F1) and second (F2) orders, while in depolymerization in the presence of γ-Al_2_O_3_ and without a catalyst, the process was described by diffusion models such as the Jander equation (D3) and Zhuravlev, Lesokin, Tempelman equation (D5).

## 1. Introduction

Polymers based on lactide and glycolide are a well-known class of biodegradable polymers that have recently become attractive objects of both basic and applied research. Due to their special properties, such polymers are used in light and food industry as a material for the manufacture of disposable tableware and packaging [1]. Polylactide (PLA) and copolymers of glycolide and lactide are widely used in medicine as a material for the production of suture filaments [2], implants for orthopedics [3], in pharmaceuticals as a polymer shell for targeted drug delivery systems [4,5], and as one of the most common materials for 3-D printing [6]. They are obtained primarily by ring-opening polymerization of lactide and glycolide, which are synthesized in the process of catalytic depolymerization of the oligomers of the corresponding hydroxycarboxylic acids. The polymerization of cyclic diesters has been studied in considerable detail, which is reflected in numerous review articles that include information about the used catalysts and the conditions of the process [7,8]. At the same time, depolymerization of polymers and oligomers of glycolic (GAO) and lactic (LAO) acids is of no less importance as a method for the production of glycolide and lactide, respectively, the mechanism of which is still being discussed. Significant contributions to PLA depolymerization research were made by Nishida and coworkers, who showed the process features in the presence of various oxide catalysts [9]. They suggested mechanisms of PLA depolymerization, among which the intramolecular mechanism (backbiting or unzipping depolymerization) was considered the most preferable in terms of lactide formation. Mention of intramolecular mechanism was also found in other studies related to the development of methods for the synthesis of lactide [10,11,12,13]. Recently, methods of isoconversional thermal analysis were widely used to study the kinetics of PLA depolymerization in the presence of various inorganic catalysts, such as zeolites, ZnO, TiO_2_, and others [14,15,16,17,18,19,20,21]. In these studies, the obtained kinetic data were compared with known kinetic models [22] and the most suitable depolymerization mechanism was determined. It should be noted that all these studies concern the processing of PLA into lactide. No less important is the process of depolymerization of low-molecular weight oligomers of hydroxycarboxylic acids as the main industrial method for the obtainment of cyclic diesters, the mechanism of which has not been practically studied. Kameno et al. considered the features of non-catalytic depolymerization of LAO [23]. Using isoconversional thermal analysis, it was shown that the depolymerization of oligomers involves two parallel stages, the first of which was controlled by diffusion and had an activation energy of about 89.9 kJ/mol (unzipping depolymerization or intermolecular transesterification). The second stage had a higher activation energy of 156.5 kJ/mol, which the authors attribute to formation of oligomer bubbles during the reaction. At the same time, it is interesting to study the kinetics of catalytic depolymerization of oligomers, as well as to consider the nature of their interactions in the reaction process to improve the technology of synthesis of cyclic diesters and polymers based on them.

**Figure 1:**
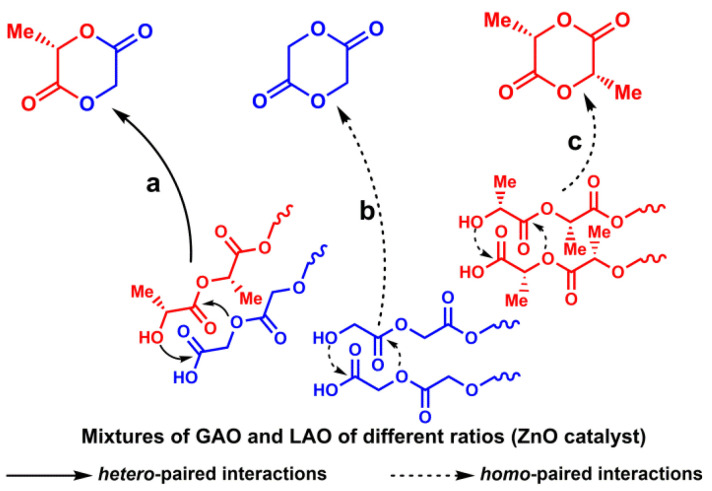
Intermolecular depolymerization of mechanical mixtures of GAO:LAO: *hetero*-paired interactions (**a**), *homo*-paired interactions (**b**,**c**).

Earlier, we showed by the example of mechanical mixtures of GAO and LAO that along with intramolecular interactions, intermolecular interactions of oligomers in the form of paired associates on the ZnO surface can also occur (Figure 1) [24]. One of the main products of intermolecular depolymerization is 3-methylglycolide (3-MG) (Figure 1a), which contains both glycolic and lactic acid moieties in its structure. Other products of depolymerization are glycolide (Figure 1b) and lactide (Figure 1c), which are formed both during intermolecular and intramolecular interactions of oligomeric molecules.

This work was devoted both to the study of depolymerization kinetics of low molecular weight oligomers of glycolic and lactic acids in the presence of ZnO, γ-Al_2_O_3_ and without a catalyst by isoconversional thermal analysis, and to the determination of the nature of the interactions of oligomeric molecules, depending on the nature of the active sites of the catalysts.

## 2. Materials and Methods

### 2.1. Materials

All reagents and solvents were used as received from the commercial vendors without further purification. Water solutions of l-lactic (Corbion, 80 wt.%, Gorinchem, Netherlands) and glycolic (Acros Organics, 70 wt.%, Geel, Belgium) acids were used as initial substances for the synthesis of the LAO and GAO. ZnO and γ-Al_2_O_3_ were used as catalysts of depolymerization. Commercial ZnO (Vekton, St. Petersburg, Russia) was calcined at 400 °C for 3 h before the experiments. γ-Al_2_O_3_ was obtained by dehydration of pseudoboehmite (KNT group, Sterlitamak, Russia) at 600 °C for 2 h.

### 2.2. Synthesis of GAO and LAO

Calculated amounts of aqueous hydroxycarboxylic acid solutions were placed in a rotary evaporator flask of the Heidolph Hei-VAP Advantage apparatus (Heidolph Instruments GmbH & CO. KG, Schwabach, Germany). The syntheses were carried out with a temperature increase from 130 to 180 °C and pressure decrease from 375 to 75 mm Hg for 5 h [25,26]. Obtained GAO and LAO were used for depolymerization experiments (Section 2.3) and kinetic study (Section 2.6).

### 2.3. Depolymerization of GAO:LAO Mixtures

On completion of the syntheses, six batches of oligomers mixtures, namely GAO:LAO = 3:1 (samples A and D); 1:1(samples B and E) and 1:3 (samples C and F), 50 grams of each were put into six reaction flasks for further depolymerization in the presence of γ-Al_2_O_3_ (samples A, B, C) and without catalyst (samples D, E, F). A glass reactor, consisting of a reaction flask with a thermometer, a distillation adapter, and a receiving flask, was used for depolymerization of the obtained oligomer mixtures (Supplementary Materials, Figure S1). The 1 wt.% of depolymerization catalyst was added to the samples A, B, C. Samples D, E, and F were depolymerized without the catalyst. All components of the mixtures were thoroughly homogenized by heating about 100–120 °C. Depolymerization of GAO:LAO mixtures in the presence of ZnO was described in our previous study [24].

The depolymerization of the mixtures was carried out at 220 °C and 15–30 mm Hg. Upon completion of the syntheses, the yields of the depolymerization products (samples A*, B*, C*, D*, E*, F*, their identification presented in Supplementary Materials, Figures S2–S13) were determined and their identifications were carried out. Model depolymerization reactions (Section 3.2) of GAO:LAO mixture (1:1) were carried out in the same conditions in the presence of ZnO. The duration of depolymerization was varied for modeling of different conversion.

### 2.4. Characterization of Catalysts

The phase composition of the depolymerization catalyst was studied using X-ray diffraction analysis (XRD) on the Shimadzu XRD-6000 (Shimadzu Corporation, Kyoto, Japan) with CuKα radiation (γ = 1.5418 Å) at the range of 10°–80° (2θ). The analysis of phase composition was carried out based on PDF 4+ database and the POWDER CELL 2.4 program (developed by Dr. W. Kraus and Dr. G. Nolze, Berlin, Germany).

The specific surface area of depolymerization catalysts was studied using low-temperature N_2_ adsorption (−196 °C) on an automatic gas-adsorption analyzer TriStar 3020 (Micromeritics, Norcross, GA, USA) by the BET method.

Acid-base properties of the catalyst surface were studied by IR spectroscopy of probe molecules of pyridine and CDCl_3_ for the determination of acidic and basic active sites, respectively, on Fourier spectrometer Shimadzu IR Tracer-100 (Shimadzu Corporation, Kyoto, Japan) with a resolution of 4 cm^−1^ and a spectral accumulation number of 100.

### 2.5. Characterization of GAO, LAO, and Their Depolymerization Products

Molecular weights and molecular weight distributions of GAO and LAO were determined by gel permeation chromatography in the liquid Agilent 1200 chromatograph (Agilent Technologies, Santa Clara, CA, USA) with a refractometric detector (chloroform (LAO) and chloroform:hexafluoroisopropanol (GAO), a flow rate of 1 mL/min). Calibration was performed with polystyrene standards.

The structures of obtained oligomers and depolymerization products were stated using ^1^H and ^13^C NMR, GC, and GC/MS. ^1^H and ^13^C NMR spectra were recorded on Bruker AVANCE III HD spectrometer (Bruker Corporation, Billerica, MA, USA) with an operating frequency of 400 MHz and 100 MHz at 25 °C, solvent—CDCl_3_, TMS as the internal standard.

The GC analysis was performed on the Maestro-2 chromatograph (Interlab, Moscow, Russia) equipped with Agilent Cyclosil-B Chiral Column (30 m × 0.25 mm × 0.25 μm, He as a carrier, flow rate of 1 mL/min) (Agilent Technologies, Santa Clara, CA, USA). Determination of the depolymerization products was carried out using standards and GC/MS (Agilent 6890 N chromatograph with mass spectral detector Agilent 5973 N, Agilent Technologies, Santa Clara, CA, USA).

### 2.6. Study of Kinetic of GAO:LAO Depolymerization

#### 2.6.1. Preparation of the Samples

Samples for kinetics studies were prepared in the form of thin films obtained by the dissolution of oligomers in hexafluoroisopropanol and dispersion of the catalyst in solution with vigorous stirring for 2 h. The resulting films were cast onto Petri dishes and dried in a vacuum oven at a temperature of 50 °C and a pressure of 7.5 mm Hg for 24 h.

#### 2.6.2. Kinetic Analysis Methods

The kinetics of depolymerization of GAO:LAO mixture was determined based on thermogravimetric data obtained at a heating rate of 5, 10, 15, and 20 K/min using STA 449 F1 Jupiter (NETZSCH-Gerätebau GmbH, Selb, Germany). The activation energy of the process was calculated using the Ozawa–Flynn–Wall (OFW) [27,28], Coats–Redfern (CR) [29], and Friedman (FR) [30] methods in the range of conversion of 0.1–0.9 in increments of 0.05. Using the obtained TG curves, conversion (α) was determined according to Equation (1).

(1) $$\alpha =\frac{m_{i}-m_{t}}{m_{t}-m_{e}}$$

where *m*_t_, *m*_i_, and *m*_e_ are the mass at time *t*, initial mass, and end mass of the sample

To calculate the activation energy for each heating rate, the temperature corresponding to the desired conversion was determined on the TG curves. Then, the linear dependences of 1000/*T* on logβ (OFW method, Equation (2)), 1000/*T* on ln(β/*T*^2^) (CR method, Equation (3)), and 1000/*T* on ln(dα/d*t*) (FR method, Equation (4)) for each heating rate were plotted.

(2) $$\log\beta =\log\left[\frac{A_{\alpha }\cdot E_{\alpha }}{R\cdot g\left(\alpha \right)}\right]-2.315-0.4567\cdot \frac{E_{\alpha }}{RT_{\alpha }}$$

(3) $$\ln\left(\frac{\beta }{T_{\alpha }^{2}}\right)=\ln\left[\frac{A_{\alpha }\cdot R}{E_{\alpha }\cdot g\left(\alpha \right)}\right]-\frac{E_{\alpha }}{RT_{\alpha }}$$

(4) $$\ln{\left(\frac{d\alpha }{dt}\right)}_{\alpha }=\ln\left[f(\alpha )\cdot A_{\alpha }\right]-\frac{E_{\alpha }}{RT_{\alpha }}$$

where β is a heating rate (in K/min), *g*(α) is a reaction model function in the integral form, *f*(α) is a reaction model function in the differential form.

The activation energy for each conversion was determined from the slope of the obtained line.

To determine the kinetic model of the reaction and the preexponential factor, the compensation effect was used (Equation (5)) [31].

(5) $$\ln A_{j}=aE_{j}+b$$

where *E*_j_ is an activation energy obtained by model-free FR method, *a* and *b* are the parameters of the compensation effect (*Note!* Such a form of Equation (5) is used for calculation of the parameters of the compensation effect. When the calculation of activation energy and preexponental factor of studied processes is carried out, subscripts “*j*” in Equation (5) are replaced on “α”).

To determine the parameters *a* and *b*, the FR method was used, with which, pairs of *E*_α_ and ln*A*_α_ values were calculated using a series of kinetic models, describing various types of possible interactions (Table 1). Based on the obtained data (*E*_α_ vs. ln*A*_α_), a linear relationship was built and the parameters of the compensation effect were determined. The obtained values of parameters *a* and *b* were used for calculation of pairs *E*_α_ and *A*_α_ at the defined conversion, which were then substituted into Equation (6) to determine the experimental function *f*(α).

(6) $$f\left(\alpha \right)=\beta \cdot {\left(\frac{d\alpha }{dT}\right)}_{\alpha }\cdot {\left[A_{\alpha }\cdot \exp\left(\frac{-E_{\alpha }}{RT_{\alpha }}\right)\right]}^{-1}$$

The obtained experimental function *f*(α) was compared with the known theoretical functions to determine the most suitable kinetic model of the process under study. The experimental and theoretical functions were normalized to 1.

## 3. Results and Discussion

### 3.1. Depolymerization of GAO and LAO Mixtures in the Presence of ZnO, γ-Al_2_O_3_, and Without Catalyst

The structure of obtained GAO and LAO was investigated by IR and NMR spectroscopy (Supplementary Materials, Figures S14–S19). ^1^H NMR spectrum of LAO contained signals of protons of methyl groups in the region of 1.55–1.57 ppm (doublet) and methine groups in the region of 5.09–5.22 ppm (quartet). Characteristic signals in the range of 4.1–5.0 ppm were present in the ^1^H NMR spectrum of GAO. Along with the structure, the molecular weight characteristics of the oligomers were determined (Table 2).

According to the obtained data, such oligomers can be used as starting reagents in the study of mechanism of GAO and LAO depolymerization, since they have close weight average molecular weights, which, on the one hand, were optimal for the production of cyclic diesters with high yield [32], on the other hand, coincided with the properties of the oligomers used in our previous work [24].

**Figure 2:**
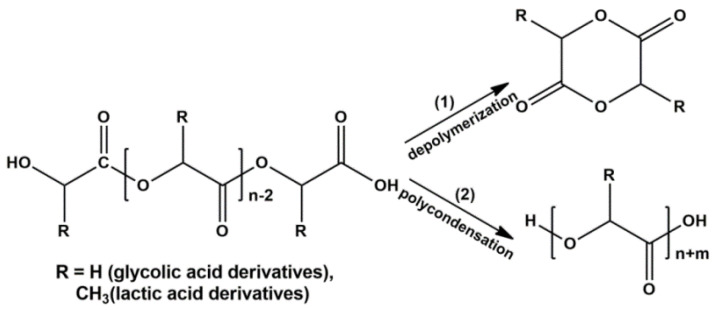
Possible reactions for GAO and LAO: depolymerization (1) and polycondensation (2).

ZnO and γ-Al_2_O_3_ were used as depolymerization catalysts as compounds having various acid-base surface properties that directly affect the synthesis of cyclic diesters. According to the XRD results, ZnO and γ-Al_2_O_3_ were single-phase catalysts and had zincite and γ-Al_2_O_3_ structures, respectively (Supplementary Materials, Figure S20 and Table S1). Other important characteristics of heterogeneous catalysts of depolymerization were the morphology and nature of their surface (Supplementary Materials, Figures S21–S23 and Table S2). An analysis of the IR spectra of oxides in the region of hydroxyl groups (3200–4000 cm^−1^) and the IR spectra of pyridine molecules adsorbed on the surface of the oxide (1540–1550 cm^−1^) showed that there were no Bronsted acid sites on the surface of the studied samples. Adsorption of CDCl_3_ revealed three types of basic sites (BS) for γ-Al_2_O_3_ and two types of BS for ZnO. The strongest and the weakest BS for ZnO and γ-Al_2_O_3_ were characterized by the bands of 2150, 2214 cm^−1^, and 2241, 2248 cm^−1^, respectively. Strong BS (BS of type I) were caused by Me–O–Me bridging oxygen atoms, and weak BS (BS of type III) were due to oxygen atoms of OH groups [33]. BS of medium strength (BS of type II) can be other oxygen atoms bonded to a metal atom in the tetrahedron or octahedron of the crystal structure of the oxide, as well as oxygen atoms of other OH groups of oxides. As we expected, ZnO naturally had stronger BS compared to γ-Al_2_O_3_, but the maximum concentration of BS on the surface of γ-Al_2_O_3_ was observed. An analysis of the IR spectra of oxides with adsorbed pyridine molecules, which contain two characteristic bands at 1450–1452 cm^−1^ and 1611–1620 cm^−1^, showed that when passing from γ-Al_2_O_3_ to ZnO, a decrease in the amount of Lewis acid sites (LAS) on the oxide surface was observed. At the same time, the strongest LAS were presented on the surface of γ-Al_2_O_3_ in comparison with ZnO because of the difference at one of characteristic bands (1620 vs. 1611 cm^−1^; increase in wavenumber of the band indicated the increase of the strength of LAS) [34]. However, when assigning the number of BS and LAS to the surface of the studied oxides, the largest number of sites was contained on the ZnO surface. Thus, the difference in the acid-base properties of the surface of the used oxides mainly in the strength of active sites of the catalyst, making it possible to evaluate their role on the depolymerization of hydroxycarboxylic acid oligomers. Depolymerization of oligomers may be accompanied by their subsequent polycondensation due to the presence of reactive terminal hydroxyl and carboxyl groups (Figure 2). Therefore, both of these reactions should be considered during the study of depolymerization.

To assess the nature of interactions, the depolymerization of GAO:LAO mixtures of various compositions was carried out in the presence and absence of selected oxide catalysts. The quantitative composition of depolymerization products is shown in Table 3. Since 3-MG and lactide contain chiral carbon atoms, they exist as l-3-MG, d-3-MG, l-lactide, *meso*-lactide, and d-lactide.

The main products of depolymerization were glycolide, 3-MG, and lactide, with l-3-MG as the most likely product of intermolecular depolymerization, depending on the ratio of the starting oligomers, and contained in an amount of 12–38 wt.%. It is important to note that glycolide and lactide can be formed both as a result of *homo*-paired interactions of the corresponding oligomers (Figure 1b,c), and during intramolecular interactions of GAO or LAO. Side products included low molecular (di-, tri-, and tetramers) linear and cyclic oligomers, lactic and glycolic acids.

**Figure 3:**
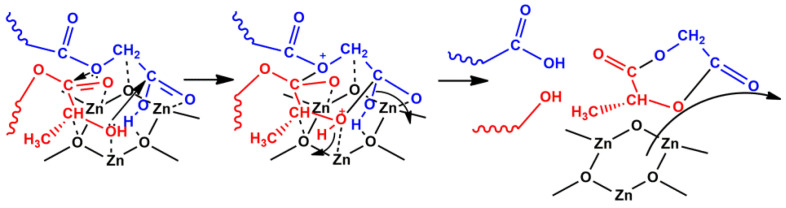
Depolymerization of GAO and LAO on the surface of ZnO.

The highest yield of crude diesters was observed in the case of depolymerization in the presence of ZnO due to several factors. On the one hand, among the used catalysts, ZnO had the largest number of BS relative to the specific surface area of catalysts (3.24 μmol/m^2^), which led to the highest yield of diesters obtained in the shortest synthesis time. On the other hand, the strength of BS and the structure of ZnO affected the yield of diesters. So, there were medium BS on its surface that catalyzed mainly the target depolymerization reaction. In addition, ZnO had a hexagonal modification of zincite, which probably contributed to the formation of six-membered diester cycles due to the geometric correspondence of oligomers and active sites of the catalyst surface (Figure 3).

**Figure 4:**
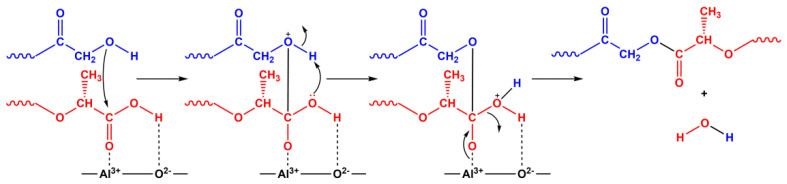
Polycondensation of GAO and LAO on the LAS of γ-Al_2_O_3_.

The decrease in the yield of diesters upon depolymerization in the presence of γ-Al_2_O_3_ was because the stronger LAS of its surface catalyzed the polycondensation reaction of oligomers, which complicated the course of target depolymerization reaction due to an increase in the molecular weight of oligomers and, as a consequence, the viscosity of the reaction mixture (Figure 4).

It is important to note that depolymerization of oligomers without oxide catalysts should nevertheless be considered as catalytic. The interaction of oligomers proceeds under conditions of acid homogeneous catalysis by protons of residual hydroxycarboxylic acids or terminal groups of oligomeric molecules. The process is characterized by low yields of diesters due to the prevalence of the polycondensation over the depolymerization, which, as in the case of γ-Al_2_O_3_, leads to an increase in the molecular weight of oligomers and the viscosity of the reaction mixture.

Since chiral LAO are used along with GAO to study the nature of interactions of oligomers, depolymerization products contain other 3-MG and lactide isomers. One of the reasons for the appearance of depolymerization products along with the expected l-isomers of 3-MG and lactide of other isomers of cyclic diesters is the LAO epimerization due to the abstraction of α-hydrogen from one of the methine groups, proceeding mainly on strong BS of catalysts. As a result, the chirality of some carbon atoms changed, which led to the appearance of other optical isomers [12,24] (Supplementary Materials, Figure S24). Another reason for the formation of *meso*- and d-isomers during depolymerization of oligomers was associated with the presence of d-isomer impurities in the initial commercial l-lactic acid, which were incorporated into the structure of its oligomeric molecules and led to a change in the chirality of 3-MG and lactide [12] (Supplementary Materials, Figure S25).

The obtained regularities were in good agreement with our previous results and confirmed the following points: the main driving force of intermolecular interactions of oligomeric molecules was their spatial arrangement in the crystal structure in the form of antiparallel directed chains [35,36], similar to paired associates. This arrangement as a whole is preserved during the destruction of long-range order during melting. Catalysts naturally accelerate depolymerization and contribute to an increase in the yield of reaction products due to the occurring adsorption–desorption interactions on their surface, including because of the geometric correspondence of the active sites of the catalyst and the structural elements of the oligomers. Moreover, due to the relatively large size of the oligomeric molecules and the melt viscosity, the specific surface area did not affect the yield of cyclic diesters, since the interactions occur mainly on the external surface of the catalysts equal to 2.1 and 137.3 m^2^/g for ZnO and γ-Al_2_O_3_, respectively (the external surface was about 61.8 and 76.9 % of the total specific surface area for ZnO and γ-Al_2_O_3_, respectively).

The obtained results in the course of GAO:LAO depolymerization in the presence of ZnO, γ-Al_2_O_3_, and without the catalyst allowed the determination of the role of the nature of the active sites in studied processes (depolymerization vs. polycondensation vs. epimerization) and to explain the difference in the yield of diesters in the case of three systems. Moreover, experimental data for depolymerization of GAO:LAO mixtures of different ratios were used both to choose a suitable system for further kinetic studies and subsequent comparison between each other to discuss the proposed mechanism of the reaction.

### 3.2. Isoconversional Kinetic Study of Depolymerization of GAO and LAO

To detail the interactions between GAO and LAO, the kinetic regularities of the process were determined. Among the considered systems for kinetic experiments, a mixture of GAO and LAO (1:1) was chosen as the system characterized by the highest yield of depolymerization products and the content of the product of intermolecular interactions (3-MG). Depolymerization was carried out in the presence of ZnO, γ-Al_2_O_3_, and without a catalyst.

**Figure 5:**
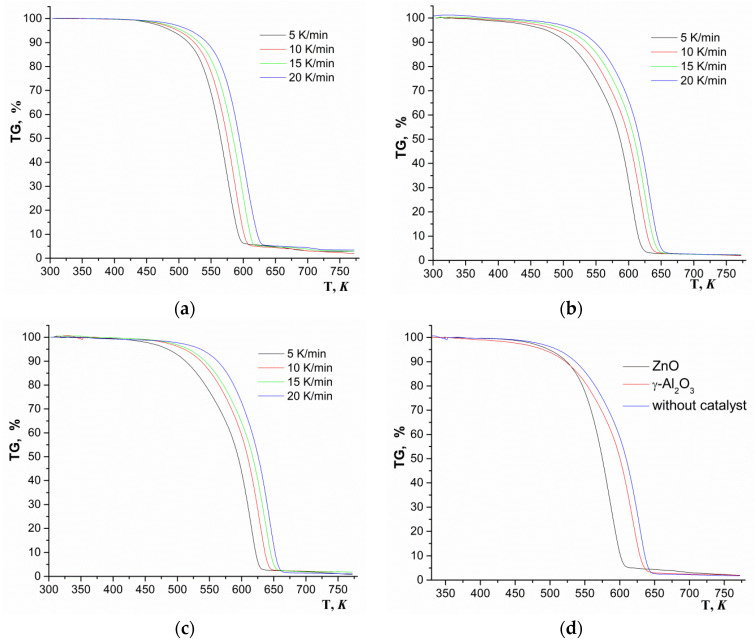
TG-curves of depolymerization of GAO:LAO mixture in the presence of catalysts and without them: ZnO (**a**), γ-Al_2_O_3_ (**b**), without catalyst (**c**), all systems at the heating rate 10 K/min (**d**).

To conduct a kinetic analysis of thermal decomposition processes in 2011, the International Confederation for Thermal Analysis and Calorimetry (ICTAC) Kinetics Committee suggested using isoconversional methods based on thermogravimetric measurements at different heating rates since these are the methods that allow the most reliable determination of the kinetics of the studied process [37]. The methods of isoconversional analysis were repeatedly used by various research groups to assess the kinetics of depolymerization of PLA and LAO [14,15,16,17,18,19,20,21,23]. The advantage of these methods is that they allow us to calculate the activation energy of the process without having an accurate understanding of its mechanism. TG curves obtained at heating rates of 5, 10, 15, and 20 K/min during depolymerization of GAO:LAO mixture in the presence of ZnO, γ-Al_2_O_3_ and without catalyst, as well as TG curves of all systems obtained at a heating rate of 10 K/min are presented in Figure 5. When comparing the TG curves of all systems obtained at a heating rate of 10 K/min (Figure 5d), it can be noted that the depolymerization of a mixture of oligomers in the presence of ZnO at the conversion of 0.1–0.9 occurred in a lower temperature range (524–600 K). Depolymerization of a mixture of oligomers in the presence of γ-Al_2_O_3_ and without a catalyst had another course of the TG curves, and the process of their depolymerization was characterized by wider temperature ranges of 524–629 K and 538–636 K, respectively.

**Figure 6:**
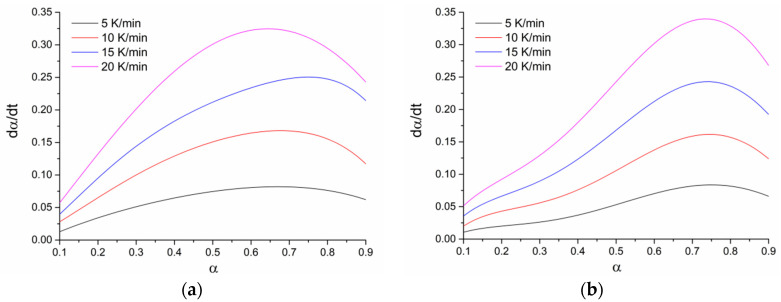

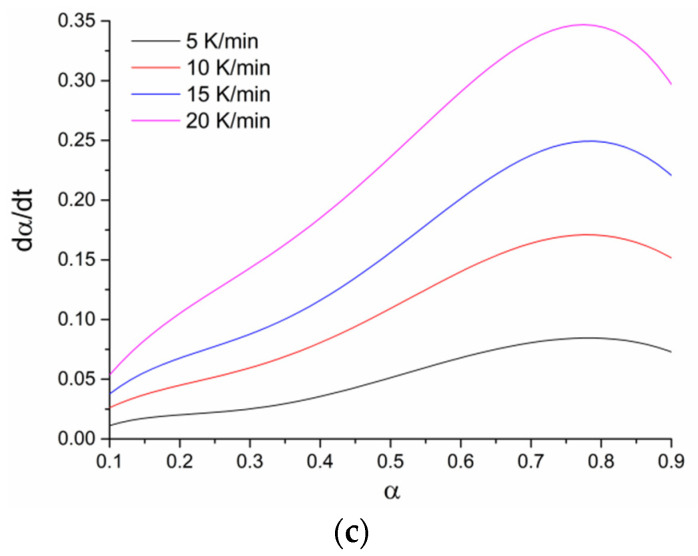
Dependence of (dα/d*t*) on oligomers conversion (0.1–0.9): ZnO (**a**), γ-Al_2_O_3_ (**b**), without catalyst (**c**).

The wider temperature range of depolymerization for the conversion of 0.1–0.9 in the case of syntheses in the presence of γ-Al_2_O_3_ and without a catalyst was due to side parallel reactions of subsequent polycondensation of oligomers (Figure 2(2)). Dependences (dα/d*t*) on the conversion (0.1–0.9) for all studied systems are presented in Figure 6. Unlike the system with ZnO, for systems with γ-Al_2_O_3_ and without a catalyst, the dependencies contained a shoulder in the region of low conversion, which may indicate several different processes during depolymerization.

**Figure 7:**
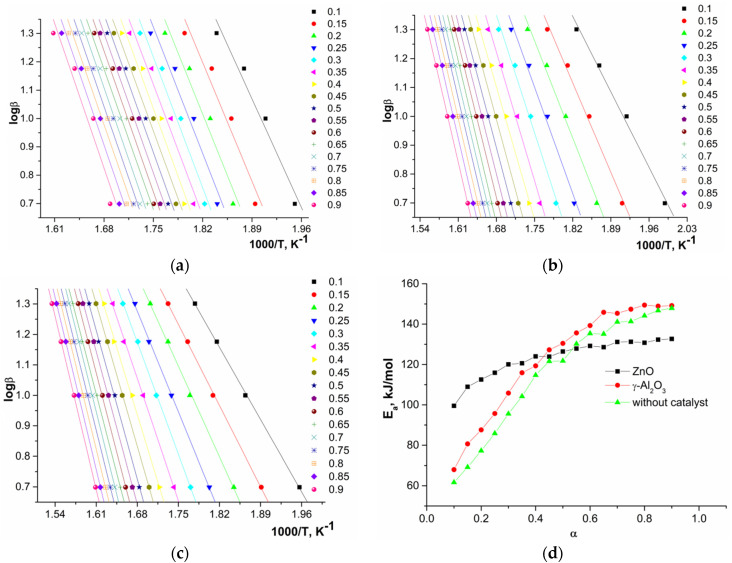
Dependences of logβ on 1000/*T* for depolymerization in the presence of ZnO (**a**), γ-Al_2_O_3_ (**b**), without catalyst (**c**), and of activation energy on conversion (**d**) calculated by OFW method.

**Figure 8:**
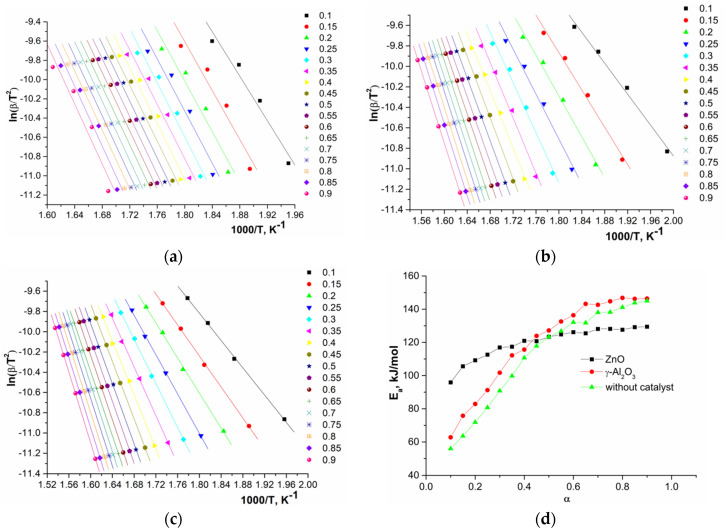
Dependences of ln(β/*T*^2^) on 1000/*T* for depolymerization in the presence of ZnO (**a**), γ-Al_2_O_3_ (**b**), without catalyst (**c**), and of activation energy on conversion (**d**) calculated by CR method.

**Figure 9:**
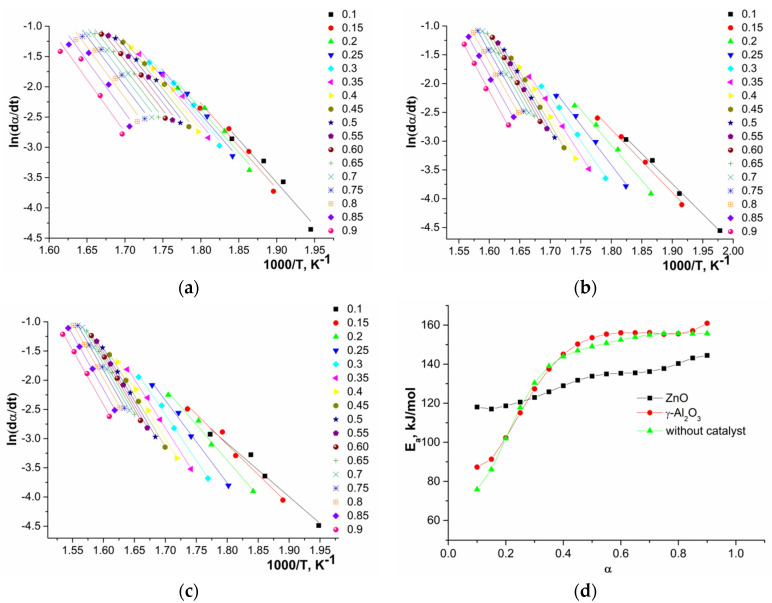
Dependences of ln(dα/d*t*) on 1000/*T* for depolymerization in the presence of ZnO (**a**), γ-Al_2_O_3_ (**b**), without catalyst (**c**), and of activation energy on conversion (**d**) calculated by FR method.

The linear dependences of 1000/T at various heating rates (logβ, ln(β/*T*^2^), and ln(dα/d*t*)) and the activation energy calculated by the OFW, CR, and FR methods are presented in Figure 7, Figure 8 and Figure 9.

An analysis of the obtained data showed that, in the first approximation, during depolymerization of a system with ZnO one stage and for systems with γ-Al_2_O_3_ and without a catalyst in certain ranges of conversion, two parallel stages or processes (two sections on the curves) were observed. The calculated average values of activation energies depending on conversion are presented in Table 4.

The observed dependences of the activation energy on conversion of oligomers during depolymerization of GAO:LAO mixture for all three systems should be considered from the point of view of simultaneously occurring reactions of depolymerization of oligomers and their subsequent polycondensation (Figure 2). As already noted, both of these reactions depend substantially on the nature of active sites of the catalysts. At oligomer conversion of 0.1–0.4, systems with γ-Al_2_O_3_ and without a catalyst were characterized by lower activation energies compared to a system with ZnO, since at lower temperatures the polycondensation reaction proceeds on the LAS of γ-Al_2_O_3_ and due to protons of oligomers carboxylic end groups. The obtained activation energies of about 60–120 kJ/mol for systems with γ-Al_2_O_3_ and without a catalyst at the conversion of 0.1–0.4 were close to published data on the kinetics of LAO polycondensation [38,39]. Close activation energies for these two systems, one of which is nominally catalytic and the other non-catalytic, was due to the fact that depolymerization of the GAO:LAO mixture in the case of a system without a catalyst should still be considered as catalytic, namely, proceeding under conditions of homogeneous acid catalysis by protons of the carboxylic end groups of oligomers. In the case of the system GAO:LAO with ZnO, the surface of which contains mainly BS, the depolymerization proceeded to a greater extent, which was characterized by high activation energies at the conversion of 0.1–0.4 and the absence of a pronounced second region (process) on the curve. We can make an assumption about the limiting stage of the processes under consideration from the obtained data. Due to high viscosity of the reaction mixture at conversion less than 0.4, the process was controlled by diffusion in the case of all systems [23]. Moreover, diffusion processes predominate for systems with γ-Al_2_O_3_ and without a catalyst, since an increase in the molecular weight due to polycondensation at the LAS made it difficult to remove the resulting cyclic diesters. With an increase of conversion (more than 0.4), the viscosity of the system decreases, and the process was controlled by a chemical reaction, which was characterized by large activation energies for all three systems.

For a system with ZnO at the conversion of 0.1–0.4, both *homo*-paired intermolecular, intramolecular interactions of GAO and LAO, and *hetero*-paired interactions were realized along with polycondensation to a lesser extent. At high conversion (0.45–0.9) small changes in the activation energy were observed, which indicated the occurrence of interactions of the same nature. The predominance of *hetero*-paired interactions with a conversion of more than 0.4 was associated with a decrease in the viscosity of reaction mixture due to a decrease in the molecular weight of oligomers, which significantly facilitated the formation of *hetero*-paired associates between GAO and LAO. In case of depolymerization of a mixture of GAO:LAO in the presence of γ-Al_2_O_3_ and without catalyst, the appearance of the second region on the curve was associated with parallel processes of polycondensation of oligomers at the LAS of oxides and due to protons of the terminal groups of GAO or LAO, which contributed to an increase in the molecular weight of oligomers. In turn, increasing molecular weight complicated the depolymerization of oligomers, which were characterized by higher activation energies in comparison with ZnO, and, as a result, led to a decrease in the yields of cyclic diesters, which was already shown.

**Figure 10:**
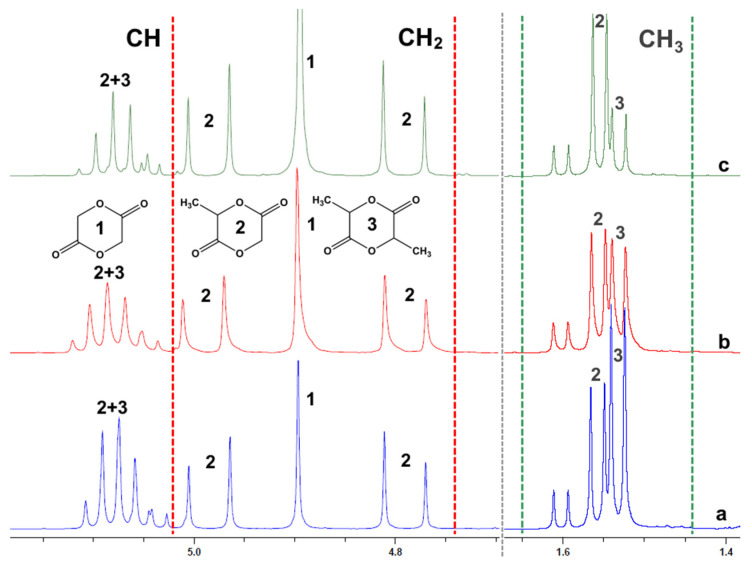
^1^H NMR spectra of main products of GAO:LAO mixture depolymerization: conversion—0.3 (**a**), 0.6 (**b**), 0.75 (**c**).

To confirm the assumption that *hetero*-paired intermolecular interactions of oligomers prevail (in the case of high conversion) during their depolymerization, model depolymerization of GAO:LAO mixture (1:1) was carried out in the presence of ZnO. Analysis of the main depolymerization products (cyclic diesters) by ^1^H NMR spectroscopy is presented in Figure 10.

At a low conversion (0.2–0.25), glycolide (a singlet from protons of the CH_2_ groups at 4.9 ppm) and lactide (a doublet from protons of the CH_3_ groups in the range of 1.52–1.55 ppm) as the products of *homo*-paired intermolecular or intramolecular interactions prevailed in them compared with 3-MG (doublet from protons of CH_3_ groups in the region of 1.55–1.58 ppm, split singlet from protons of CH_2_ groups in the region of 4.75–5.0 ppm), which was a product of *hetero*-paired intermolecular interactions. With an increase in the conversion of oligomers, an increase in the content of 3-MG was observed due to a decrease in the viscosity of the reaction mixture because of a decrease in the molecular weight of oligomers, which significantly facilitated the formation of *hetero*-paired associates between GAO and LAO.

To determine the most suitable kinetic model describing the depolymerization of GAO and LAO, it was necessary to determine the form of the experimental function *f*(α) for all three systems and compare it with the known kinetic models [22]. To calculate the experimental function *f*(α) and search for a suitable kinetic model, the compensation effect was used. For systems with γ-Al_2_O_3_ and without catalyst, the parameters were calculated in the ranges of conversion of 0.1–0.4 and 0.45–0.9. The obtained values of the compensation parameters are presented in Table 5.

**Figure 11:**
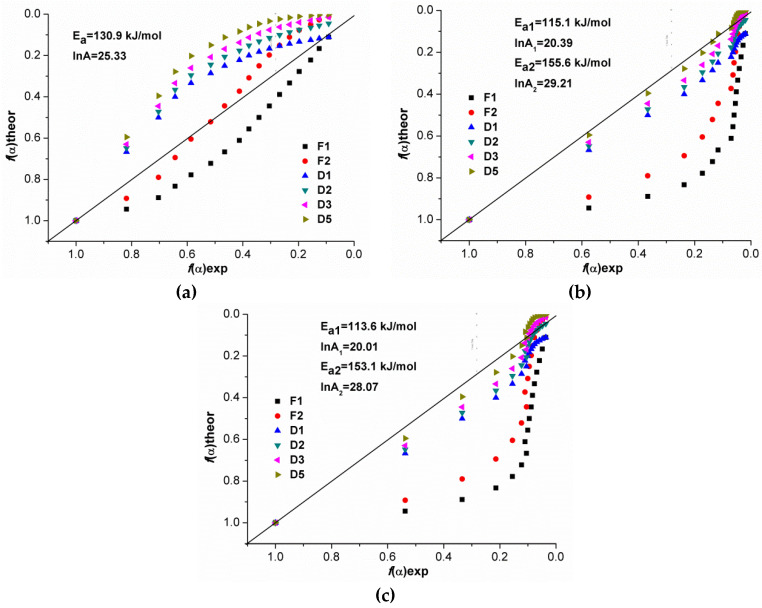
Comparison of experimental and theoretical *f*(α) for depolymerization in the presence of ZnO (**a**), γ-Al_2_O_3_ (**b**), and without catalyst (**c**) at 5 K/min.

The calculated values of the parameters *a* and *b* were used to determine the experimental function *f*(α). The graphs of the calculated experimental functions *f*(α) and theoretical models for all studied systems at heating rates 5 K/min are presented in Figure 11 (all heating rates in Supplementary Materials, Figures S26–S28).

When comparing the experimental and theoretical functions *f*(α), it was noted for all three systems that as the heating rate changed the form of the experimental curves, as expected, it changed insignificantly. The experimental function *f*(α) for a system with ZnO lies in the region of the first (F1) and second (F2) order kinetic models, which confirmed our assumptions about the mixed nature of the interactions of GAO and LAO. The experimental curve was shifted towards diffusion models at the conversion of 0.1–0.4 for the same system since at the initial stage, viscosity of the system was high and therefore the process was controlled by diffusion. With an increase of conversion, depolymerization was limited by a chemical reaction.

The experimental functions *f*(α) for systems with γ-Al_2_O_3_ and without a catalyst were similar and significantly differed from *f*(α) for a system with ZnO. The experimental functions were in the region of diffusion kinetic models, mainly models D3 and D5 for both systems. The similarity with diffusion models can be attributed to the significant contribution of the subsequent polycondensation reaction proceeding in parallel, leading to an increase in the molecular weight of oligomers and the viscosity of the system, which complicated the removal of the formed cyclic diesters. Higher values of the activation energy of depolymerization of oligomers in the presence of γ-Al_2_O_3_ and without catalyst were also associated with the occurring polycondensation. Comparing the obtained kinetic regularities with similar systems presented in the literature, it should be noted that in them, as in the present work, in most cases the experimental kinetic models were more similar to the diffusion models (D3, D5) [19,21,23]. In the case of the kinetic models presented in the literature, diffusion as a limiting stage was most probable, since PLA had a molecular weight of more than 10,000, which had a higher melt viscosity than low molecular weight LAO and undergo depolymerization. It should be noted that obtained kinetic regularities were in good agreement with experimental data of depolymerization of GAO:LAO mixtures (Section 3.1). They explained the role of the nature of active sites of catalysts in oligomers interactions and their influence on the main (depolymerization) and side (polycondensation and epimerization) reactions that proceeded during the synthesis of diesters.

**Figure 12:**
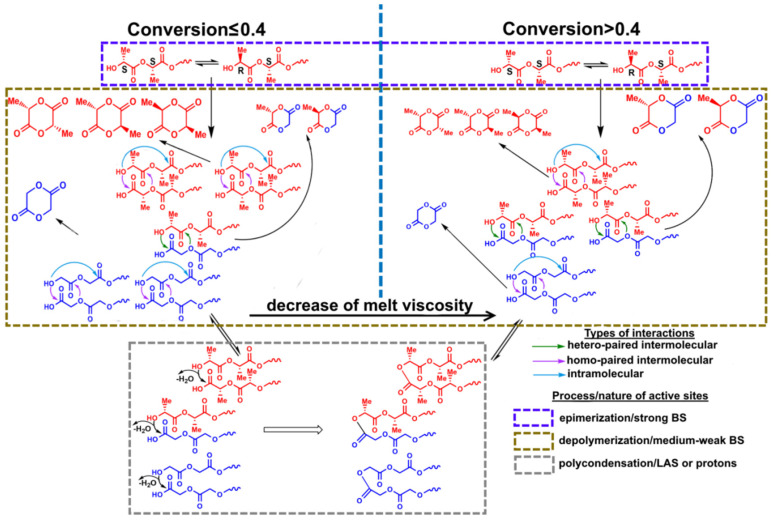
Detailed scheme of depolymerization for an example of GAO:LAO mixture.

Thus, taking into account all the experiments performed, the depolymerization of oligomers of hydroxycarboxylic acids using the example of GAO:LAO mixture can be described as a detailed scheme shown in Figure 12. In the synthesis of cyclic diesters from the corresponding oligomers of hydroxycarboxylic acids, in addition to the main depolymerization reaction (medium and weak BS), in which, depending on the conversion, mixed or intermolecular interactions predominated, side reactions of epimerization (strong BS) and polycondensation occurred (LAS or protons of the carboxylic end groups of oligomers). Consequently, the nature of active sites of the catalysts and their amount affected the depolymerization of oligomers. The obtained regularities have a wide practical application. They can be used in the development of highly selective heterogeneous catalysts both for the synthesis of cyclic diesters of hydroxycarboxylic acids and recycling of lactic- and glycolic acid-based biodegradable polymers into valuable organic compounds.

## 4. Conclusions

An experimental study of the nature of the interactions of oligomers with the example of GAO:LAO mixtures by NMR spectroscopy and gas chromatography showed that depolymerization produces l-3-MG and d-3-MG as products of *hetero*-paired intermolecular interactions along with glycolide and lactide isomers (l-, *meso*- and d-), which can form both in *homo*-paired intermolecular interactions and in intramolecular interactions. Moreover, under the conditions of model depolymerization of the GAO:LAO mixture, it was noted that with an increase of conversion, *hetero*-paired intermolecular interactions predominated due to a decrease in the molecular weight and viscosity of the reaction mixture, which greatly facilitated the formation of *hetero*-paired associates between GAO and LAO.

The yield of cyclic diesters was substantially affected by the nature of the active sites of the catalysts. The highest yield of crude diesters was obtained during the depolymerization of oligomers in the presence of ZnO, which, in comparison with γ-Al_2_O_3_, had the highest number of BS relative to the specific surface area of the catalysts (3.24 μmol/m^2^). The diester yield was also affected by the BS strength and the ZnO structure, which contained medium BS on its surface that catalyzed the predominantly depolymerization reaction, and had a hexagonal modification of zincite, which probably contributed to the formation of six-membered diesters due to the geometric correspondence of oligomers and active sites of the catalyst surface. The depolymerization of GAO:LAO mixtures in the presence of γ-Al_2_O_3_ and without a catalyst was characterized by lower yields of crude diesters due to the prevalence of the side polycondensation reaction occurring both at the LAS and the protons of the end groups of oligomers. The polycondensation of oligomers led to an increase in its molecular weight and the viscosity of the reaction mixtures.

To detail the interactions of oligomers during their depolymerization in the presence of ZnO, γ-Al_2_O_3_, and without a catalyst for GAO:LAO mixture (1:1), kinetic regularities of the process were determined by isoconversional analysis (OFW, CR, and FR methods) based on thermogravimetric data. A nonmonotonic change in the activation energy depending on conversion indicated several parallel stages, namely depolymerization, and polycondensation. During the depolymerization of the GAO:LAO mixture in the presence of ZnO, the contribution of polycondensation was insignificant, since BS predominated on its surface, catalyzing mainly depolymerization, which was characterized by a change in the activation energy in the range of 120–140 kJ/mol. In the case of the depolymerization of the GAO:LAO mixture in the presence of γ-Al_2_O_3_ and without a catalyst, lower activation energies at the conversion of 0.1–0.4 and its large values at the conversion of 0.45–0.9 in comparison with ZnO were associated with parallel processes of polycondensation of oligomers. When determining the kinetic model for the studied systems, it was shown that in the case of depolymerization with ZnO, the experimental curve *f*(α) lay in the region of theoretical curves of the first (F1) and second (F2) orders, which indicated a mixed nature of the interaction of oligomers. The experimental *f*(α) curves for systems with γ-Al_2_O_3_ and without catalyst were similar to each other and are in the region of diffusion models (D3 and D5).

## Figures and Tables

**Table 1:** Some of the kinetic models used in calculation of kinetic of depolymerization.

No.	Reaction Model	Code	*f*(α)	g(α)
1	Avrami–Erofeyev	A2	$2\left(1-\alpha \right){\left[-\ln\left(1-\alpha \right)\right]}^{1/2}$	${\left[-\ln\left(1-\alpha \right)\right]}^{1/2}$
2	First order	F1	1−α	−ln(1−α)
3	Second order	F2	${\left(1-\alpha \right)}^{2}$	[1/(1−α)]−1
4	1-D diffusion	D1	$1/2\alpha^{-1}$	$\alpha^{2}$
5	2-D diffusion	D2	${\left[-\ln\left(1-\alpha \right)\right]}^{-1}$	(1−α)ln(1−α)+α
6	3-D diffusion (Jander)	D3	$3/2{\left(1-\alpha \right)}^{2/3}{\left[1-{\left(1-\alpha \right)}^{1/3}\right]}^{-1}$	${\left[1-{\left(1-\alpha \right)}^{1/3}\right]}^{2}$
7	3-D diffusion (Zhuravlev, Lesokin, Tempelman)	D5	$3/2{\left(1-\alpha \right)}^{4/3}{\left[{\left(1-\alpha \right)}^{-1/3}-1\right]}^{-1}$	${\left[{\left(1-\alpha \right)}^{-1/3}-1\right]}^{2}$
8	Contracting cylinder	R2	$2{\left(1-\alpha \right)}^{1/2}$	$1-{\left(1-\alpha \right)}^{1/2}$
9	Contracting sphere	R3	$3{\left(1-\alpha \right)}^{2/3}$	$1-{\left(1-\alpha \right)}^{1/3}$

**Table 2:** Molecular weight and molecular weight distribution of GAO and LAO.

Type of Oligomer	*M*_w_	*M*_n_	*D*
LAO	1400	1000	1.4
GAO	1100	600	1.8

**Table 3:** Yield and composition of products of depolymerization of GAO:LAO mixtures.

Catalyst	GAO:LAO	*τ*, min ^a^	Yield ^b^, %	Content of Depolymerization Products, wt.%						
				1 ^c^	2	3	4	5	6	7
ZnO [24]	3:1	50	90.2	47.45	29.58	3.35	12.52	1.58	0.23	5.29
	1:1	46	92.6	25.41	38.42	3.68	25.29	3.10	0.25	3.85
	1:3	45	91.3	8.26	36.21	0.37	38.53	6.80	0.62	9.21
γ-Al_2_O_3_	3:1	165	42.6	21.46	24.58	11.45	32.30	6.15	1.96	2.10
	1:1	170	74.2	14.58	30.34	10.37	34.31	7.02	0.88	2.50
	1:3	167	55.6	4.97	22.09	7.48	48.03	12.46	2.27	2.71
–	3:1	215	40.8	27.62	20.58	16.24	21.08	7.67	3.36	3.45
	1:1	220	36.2	9.31	18.94	13.53	32.35	13.31	6.25	6.31
	1:3	222	41.0	2.34	12.41	7.46	45.10	18.87	9.22	4.60

**^a^** τ—reaction time, **^b^** total yield of depolymerization products, **^c^** 1—glycolide, 2—l-3-MG, 3—d-3-MG, 4—l-lactide, 5—*meso*-lactide, 6—d-lactide, 7—side products.

**Table 4:** Activation energy values of GAO:LAO mixture depolymerization (for α—0.1–0.9).

Catalyst	${\bar{E}}_{a1},\;\mathrm{kJ}/\mathrm{mol}\;{\;}^{a}\;(\alpha )$	${\bar{E}}_{a2},\;\mathrm{kJ}/\mathrm{mol}\;\;(\alpha )$	${\bar{E}}_{a\Sigma },\;\mathrm{kJ}/\mathrm{mol}$
ZnO	123.3/120.1/130.9 (0.1–0.9)	–	123.3/120.1/130.9
γ-Al_2_O_3_	96.1/91.7/115.1 (0.1–0.4)	141.9/139.0/155.6 (0.45–0.9)	123.0/119.5/139.0
–	86.9/81.9/113.6 (0.1–0.4)	136.5/133.8/153.1 (0.45–0.9)	116.1/112.4/136.8

**^a^** E_a_ were calculated by OFW/CR/FR methods.

**Table 5:** Parameters of compensation effect.

Catalyst	5 K/min		10 K/min		15 K/min		20 K/min	
	a	b	a	b	a	b	a	b
ZnO	0.215	−2.851	0.212	−2.171	0.208	−1.751	0.205	−1.481
γ-Al_2_O_3_	0.207/0.217	−3.465/−4.590	0.203/0.212	−2.685/−3.754	0.200/0.208	−2.226/−3.247	0.197/0.205	−1.853/−2.801
–	0.205/0.214	−3.527/−4.673	0.200/0.209	−2.666/−3.905	0.197/0.206	−2.2792/−3.5615	0.194/0.202	−1.754/−2.936

## Supplementary Materials

The following are available online at https://www.mdpi.com/2073-4360/12/10/2395/s1, Figure S1: Reactor of depolymerization, Figure S2: ^13^C NMR spectrum of depolymerization products of GAO:LAO with a ratio of 3:1 (Sample A*_γ-Al_2_O_3_), Figure S3: Chromatogram of depolymerization products of GAO:LAO with a ratio of 3:1 (Sample A*_γ-Al_2_O_3_), Figure S4: ^13^C NMR spectrum of depolymerization products of GAO:LAO with a ratio of 1:1 (Sample B*_γ-Al_2_O_3_), Figure S5: Chromatogram of depolymerization products of GAO:LAO with a ratio of 1:1 (Sample B*_γ-Al_2_O_3_), Figure S6: ^13^C NMR spectrum of depolymerization products of GAO:LAO with a ratio of 1:3 (Sample C*_γ-Al_2_O_3_), Figure S7: Chromatogram of depolymerization products of GAO:LAO with a ratio of 1:3 (Sample C*_γ- Al_2_O_3_), Figure S8: ^13^C NMR spectrum of depolymerization products of GAO:LAO with a ratio of 3:1 (Sample D*_without catalyst), Figure S9: Chromatogram of depolymerization products of GAO:LAO with a ratio of 3:1 (Sample D*_without catalyst), Figure S10: ^13^C NMR spectrum of depolymerization products of GAO:LAO with a ratio of 1:1 (Sample E*_without catalyst), Figure S11: Chromatogram of depolymerization products of GAO:LAO with a ratio of 1:1 (Sample E*_without catalyst), Figure S12: ^13^C NMR spectrum of depolymerization products of GAO:LAO with a ratio of 1:3 (Sample F*_without catalyst), Figure S13: Chromatogram of depolymerization products of GAO:LAO with a ratio of 1:3 (Sample F*_without catalyst), Figure S14: IR spectrum of LAO, Figure S15: IR spectrum of GAO, Figure S16: ^1^H NMR spectrum of LAO, Figure S17: ^13^C NMR spectrum of LAO, Figure S18: ^1^H NMR spectrum of GAO, Figure S19: ^13^C NMR spectrum of GAO, Figure S20: XRD patterns of depolymerization catalysts, Figure S21: IR spectra of oxides at the region of hydroxyl groups: ZnO (a), γ-Al_2_O_3_ (b), Figure S22: IR spectra of adsorbed pyridine on the surface of ZnO (a) and γ-Al_2_O_3_ (b), Figure S23: IR spectra of adsorbed CDCl_3_ on the surface of ZnO (a) and γ-Al_2_O_3_ (b) Figure S24: Possible route of LAO epimerization, Figure S25: Routes of *meso*- and d-lactide formation, Figure S26: Comparison of experimental and theoretical *f*(α) for depolymerization in the presence of ZnO, Figure S27: Comparison of experimental and theoretical *f*(α) for depolymerization in the presence of γ-Al_2_O_3_, Figure S28: Comparison of experimental and theoretical *f*(α) for depolymerization without catalyst, Table S1: Phase composition of depolymerization catalysts, Table S2: Surface properties of ZnO and γ-Al_2_O_3_.

## References

[1] Grumezescu A.M., Holban A.M. (2018). Handbook of Bioengineering: Biopolymers for Food Design.

[2] Kim H., Kim B.H., Huh B.K., Yoo Y.C., Heo C.Y., Choy Y.B., Park J. (2017). Surgical suture releasing macrophage-targeted drug-loaded nanoparticles for an enhanced anti-inflammatory effect. Biomater. Sci..

[3] Turnbull G., Clarke J., Picard F., Riches P., Jia L., Han F., Li B., Shu W. (2018). 3D bioactive composite scaffolds for bone tissue engineering. Bioact. Materials.

[4] Mir M., Ahmed N., Rehman A. (2017). Recent applications of PLGA based nanostructures in drug delivery. Colloids Surf. B.

[5] Khlusov I.A., Kibler E.V., Kudriavtseva V.L., Tverdokhlebov S.I., Bolbasov E.N., Botvin V.V., Latypov A.D., Gazatova N.D., Litvinova L.S., Buznik V.M. (2019). Electrospray Preparation of Biocompatible Lactide-Glycolide Copolymer Capsules with Incorporation of Interferon. Dokl. Chem..

[6] Ngo T.D., Kashani A., Imbalzano G., Nguyen K., Hui D. (2018). Additive manufacturing (3D printing): A review of materials, methods, applications and challenges. Compos. Part B.

[7] Dechy-Cabaret O., Martin-Vaca B., Bourissou D. (2004). Controlled Ring-Opening Polymerization of Lactide and Glycolide. Chem. Rev..

[8] Inkinen S., Hakkarainen M., Albertsson A.-C., Sodergard A. (2011). From Lactic Acid to Poly(lactic acid) (PLA): Characterization and Analysis of PLA and Its Precursors. Biomacromolecules.

[9] Nishida H., Thakur V.K., Thakur M.K. (2016). Depolymerization Properties of Bio-Based Polymers. Handbook of Sustainable Polymers. Structure and Chemistry.

[10] Lizundia E., Ruiz-Rubio L., Vilas J.L., Leon L.M. (2016). Towards the development of eco-friendly disposable polymers: ZnO-initiated thermal and hydrolytic degradation in poly(L-lactide)/ZnO nanocomposites. RSC Adv..

[11] Chiang M.-F., Chu M.-Z., Wu T.-M. (2011). Effect of layered double hydroxides on the thermal degradation behavior of biodegradable poly(L-lactide) nanocomposites. Polym. Degrad. Stab..

[12] Huang W., Qi Y., Cheng N., Zong T., Jiang W., Li H., Zhang Q. (2014). Green synthesis of enantiomerically pure L-lactide and D-lactide using biogenic creatinine catalyst. Polym. Degrad. Stab..

[13] Jiang B., Tantai X., Hao L., Zhang L., Sun Y., Deng L., Shi Z. (2015). Synthesis of chlorostannate (II) ionic liquids and their novel application in the preparation of highquality L-lactide. RSC Adv..

[14] Zhou Q., Xanthos M. (2009). Nanosize and microsize clay effects on the kinetics of the thermal degradation of polylactides. Polym. Degrad. Stab..

[15] Carrasco F., Gámez-Pérez J., Santana O.O., Maspoch M.L. (2011). Processing of poly (L-lactic acid)/organomontmorillonite nanocomposites: Microstructure, thermal stability and kinetics of the thermal decomposition. Chem. Eng. J..

[16] Hao Y.-H., Huang Z., Wang J.-W., Yang X.-Y., Fan X.-Y., Li Y.-I., Peng Y.-W. (2016). Improved thermal stability of poly (L-lactide) with the incorporation of zeolite ZSM-5. Polym. Test..

[17] Yuzay I.E., Auras R., Soto-Valdez H., Selke S. (2010). Effects of synthetic and natural zeolites on morphology and thermal degradation of poly (lactic acid) composites. Polym. Degrad. Stab..

[18] Carrasco F., Gámez-Pérez J., Santana O.O., Maspoch M.L. (2014). Enhanced general analytical equation for the kinetics of the thermal degradation of poly (lactic acid)/montmorillonite nanocomposites driven by random scission. Polym. Degrad. Stab..

[19] Ye Q.-Q., Huang Z., Hao Y.-H., Wang J.-W., Yang X.-Y., Fan X.-Y. (2016). Kinetic study of thermal degradation of poly(L-lactide) filled with b-zeolite. J. Therm. Anal. Calorim..

[20] Feng L., Feng S., Bian X., Li G., Chen X. (2018). Pyrolysis mechanism of Poly (lactic acid) for giving lactide under the catalysis of tin. Polym. Degrad. Stab..

[21] Wang X.J., Huang Z., Wei M., Lu T., Nong D., Zhao J., Gao X., Teng L. (2019). Catalytic effect of nanosized ZnO and TiO_2_ on thermal degradation of poly (lactic acid) and isoconversional kinetic analysis. Thermochim. Acta.

[22] Khawam A., Flanagan D.R. (2006). Solid-State Kinetic Models: Basics and Mathematical Fundamentals. J. Phys. Chem. B.

[23] Kameno N., Yamada S., Amimoto T., Amimoto K., Ikeda H., Koga N. (2016). Thermal degradation of poly (lactic acid) oligomer: Reaction mechanism and multistep kinetic behavior. Polym. Degrad. Stab..

[24] Botvin V., Pozdniakov M., Filimoshkin A. (2017). Intermolecular “zipper” type depolymerization of oligomeric molecules of lactic and glycolic acids prepacked as paired associates. Polym. Degrad. Stab..

[25] Kurzina I.A., Pukhova I.V., Botvin V.V., Davydova D.V., Filimoshkin A.G., Savkin K.P., Oskomov K.V., Oks E.M. (2015). New materials based on polylactide modified with silver and carbon ions. AIP Conf. Proc..

[26] Botvin V., Latypov A., Ponarin N., Filimoshkin A. (2019). Synthesis of glycolide by catalytic depolymerization of glycolic acid oligomers modified by polyhydric alcohols. J. Phys. Conf. Ser..

[27] Ozawa T. (1965). A new method of analyzing thermogravimetric data. Bull. Chem. Soc. Jpn..

[28] Flynn J.H., Wall L.A. (1965). A quick, direct method for the determination of activation energy from thermogravimetric data. J. Polym. Sci. B Polym. Lett..

[29] Coats A.W., Redfern J.P. (1964). Kinetic parameters from thermogravimetric data. Nature.

[30] Friedman H.L. (1967). Kinetics and gaseous products of thermal decomposition of polymers. J. Macromol. Sci. Part A Pure Appl. Chem..

[31] Vyazovkin S., Brown M.E., Gallagher P.K. (2008). Isoconversional Kinetics. The Handbook of Thermal Analysis & Calorimetry: Recent Advances, Techniques and Applications.

[32] Yoo D., Kim K.D., Doo S.L. (2006). Synthesis of Lactide from Oligomeric PLA: Effects of Temperature, Pressure, and Catalyst. Macromol. Res..

[33] Paukshtis E.A., Karakchiev L.G., Kotsarenko N.S. (1979). Investigation of proton-acceptor properties of oxide surfaces by IR spectroscopy of hydrogen-bonded complexes. React. Kinet. Catal. Lett..

[34] Velthoen M.E.Z., Nab S., Weckhuysen B.M. (2018). Probing acid sites in solid catalysts with pyridine UV-Vis spectroscopy. Phys. Chem. Chem. Phys..

[35] Lee S., Hongo C., Nishino T. (2017). Crystal Modulus of Poly (glycolic acid) and Its Temperature Dependence. Macromolecules.

[36] Wasanasuk K., Tashiro K., Hanesaka M., Ohhara T., Kurihara K., Kuroki R., Tamada T., Ozeki T., Kanamoto T. (2011). Crystal Structure Analysis of Poly (l-lactic Acid) α Form On the basis of the 2-Dimensional Wide-Angle Synchrotron X-ray and Neutron Diffraction Measurements. Macromolecules.

[37] Vyazovkin S., Burnham A.K., Criado J.M., Pérez-Maqueda L.A., Popescu C., Sbirrazzuoli N. (2011). ICTAC Kinetics Committee recommendations for performing kinetic computations on thermal analysis data. Thermochim. Acta.

[38] Achmad F., Yamane K., Quan S., Kokugan T. (2009). Synthesis of polylactic acid by direct polycondensation under vacuum without catalysts, solvents and initiators. Chem. Eng. J..

[39] Baick I.H., Luciani C.V., Park S.Y., Lim T., Choi K.Y. (2012). Kinetics of Reversible Oligomerization of L-Lactic Acid with a SnCl2·2H2O/p-Toluenesulfonic Acid Catalyst. Ind. Eng. Chem. Res..

