# Supplementary material for: Kinetic Study of Depolymerization of Lactic and Glycolic Acid Oligomers in the Presence of Oxide Catalysts
Source: Polymers (Basel). 2020 Oct 17;12(10):2395. doi: 10.3390/polym12102395 (PMC7603019; doi:10.3390/polym12102395)
Supplement: Supplementary file 1 [file polymers-12-02395-s001.pdf]

Supplementary Materials

Kinetic study of depolymerization of lactic and glycolic acid oligomers in the presence of oxide catalysts

Vladimir Botvin, Svetlana Karaseva, Victor Khasanov, Anatoly Filimoshkin

Supplementary Materials

Table of Contents

General information S3
Characterization of the depolymerization reactor S4
Experimental data S5

General information

Glycolic acid oligomers (GAO)

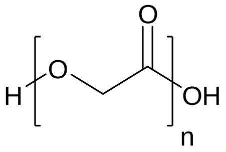

Ivory solid substance, ν_{max}/cm^{-1} (FTIR): 3511, 2959, 1755, 1414, 1160, 1084; δ_H ($CDCl_3$/HFIP mixture, 400 MHz) 4.1-5.0 (2H, m); δ_C (DMSO-d6, 100 MHz) 60.9 (CH_2), 167.7 (C=O); $\overline{M_n}$ = 600; D = 1.8.

Lactic acid oligomers (LAO)

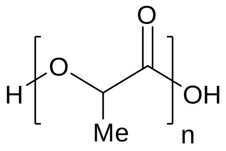

Viscous colorless substance, ν_{max}/cm^{-1} (FTIR): 3501, 2992, 2943, 1755, 1452, 1124,1082, 1040; δ_H ($CDCl_3$, 400 MHz) 1.55 (3H, d), 5.08 (1H, q); δ_C ($CDCl_3$, 100 MHz) 16.5 (CH_3), 69.0 (CH),169.8 (C=O); $\overline{M_n}$ = 1000; D = 1.4.

Glycolide

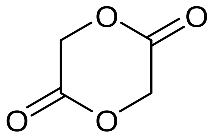

Identification in the reaction mixture: ^{1}H NMR ($CDCl_3$, 400 MHz) 4.93 (2H, s); ^{13}C NMR ($CDCl_3$, 100 MHz) 65.1 (CH_2), 165.0 (C=O); GC: 7.07 min

3-methylglycolide (3-MG)

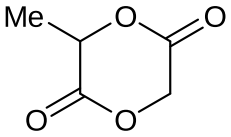

Identification in the reaction mixture: ^{1}H NMR ($CDCl_3$, 400 MHz) 1.57 (3H, d), 4.9 (2H, split s), 5.05 (1H, q); ^{13}C NMR ($CDCl_3$, 100 MHz) 15.8 (CH_3), 65.7 (CH_2), 72.0 (CH), 165.8, 167,4 (C=O); GC: 6.96 min (*l*-isomer), 6.99 (*d*-isomer).

Lactide

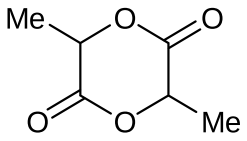

Identification in the reaction mixture: ^{1}H NMR ($CDCl_3$, 400 MHz) 1.54 (6H, d), 5.09 (3H, q); ^{13}C NMR ($CDCl_3$, 100 MHz) 15.5 (CH_3), 72.5 (CH), 168.0 (C=O); GC: 6.66 min (*meso*-isomer), 6.94 min (*l*-isomer), 7.05 min (*d*-isomer).

Characterization of the depolymerization reactor

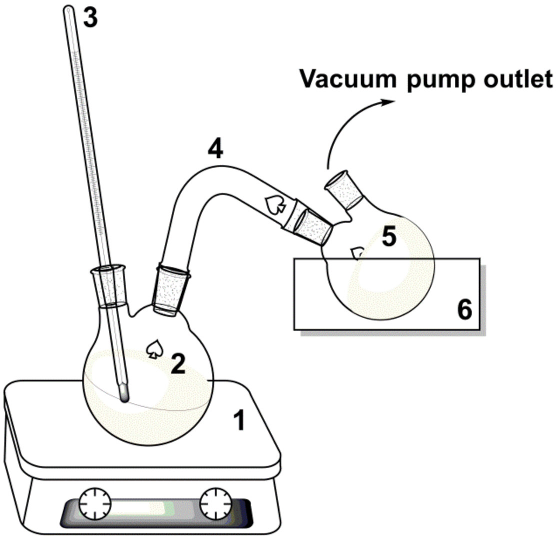

1) heating mantle, 2) reaction flask with mixed oligomers and catalyst, 3) thermometer, 4) distillation adapter, 5) receiving flask, 6) container with refrigerant (snow+$CaCl_2$)

Fig.S1. Reactor of depolymerization

Experimental data

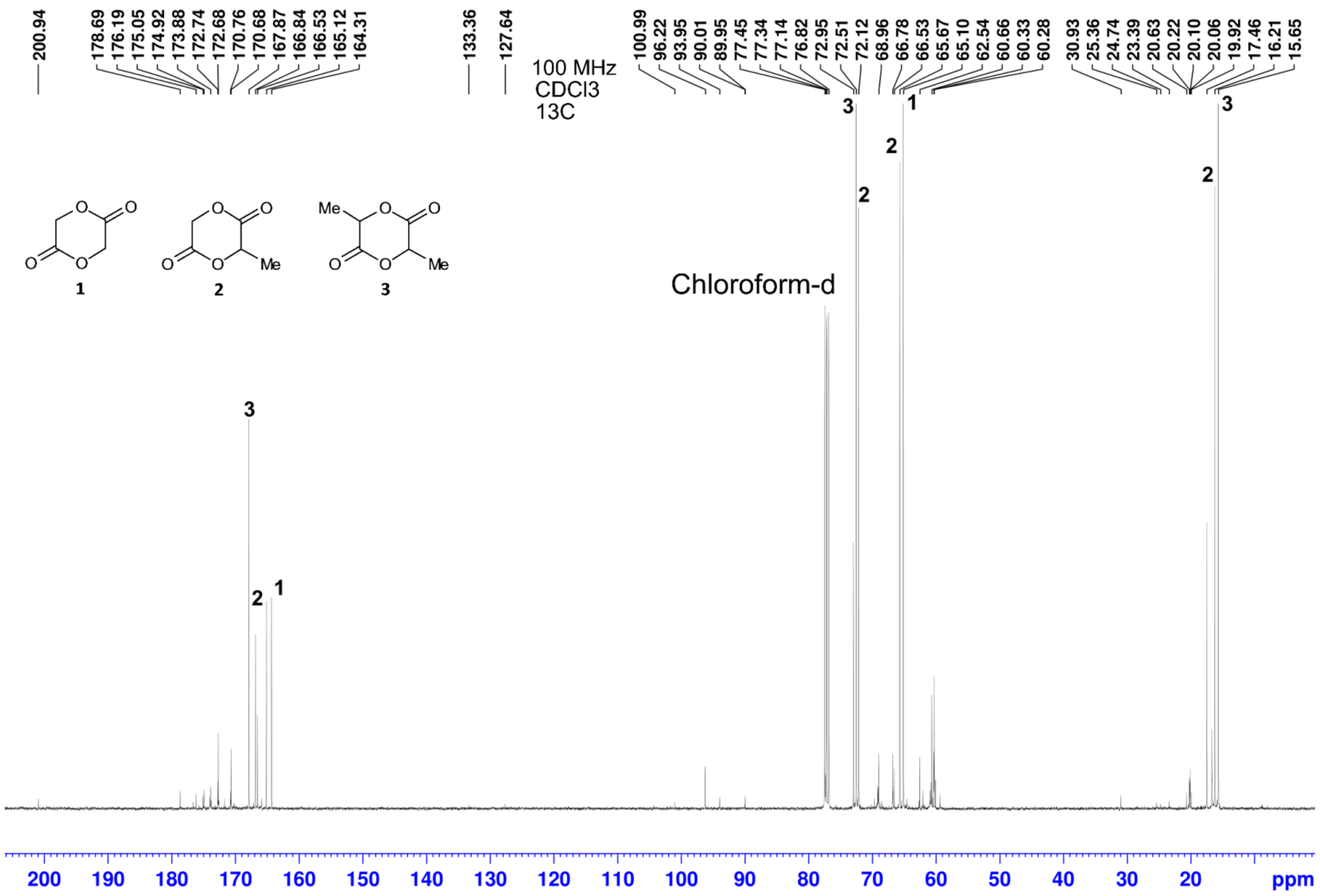

Fig. S2. ^{13}C NMR spectrum of depolymerization products of GAO:LAO with a ratio of 3:1 (Sample *A*_γ-Al$_2$O$_3$*)

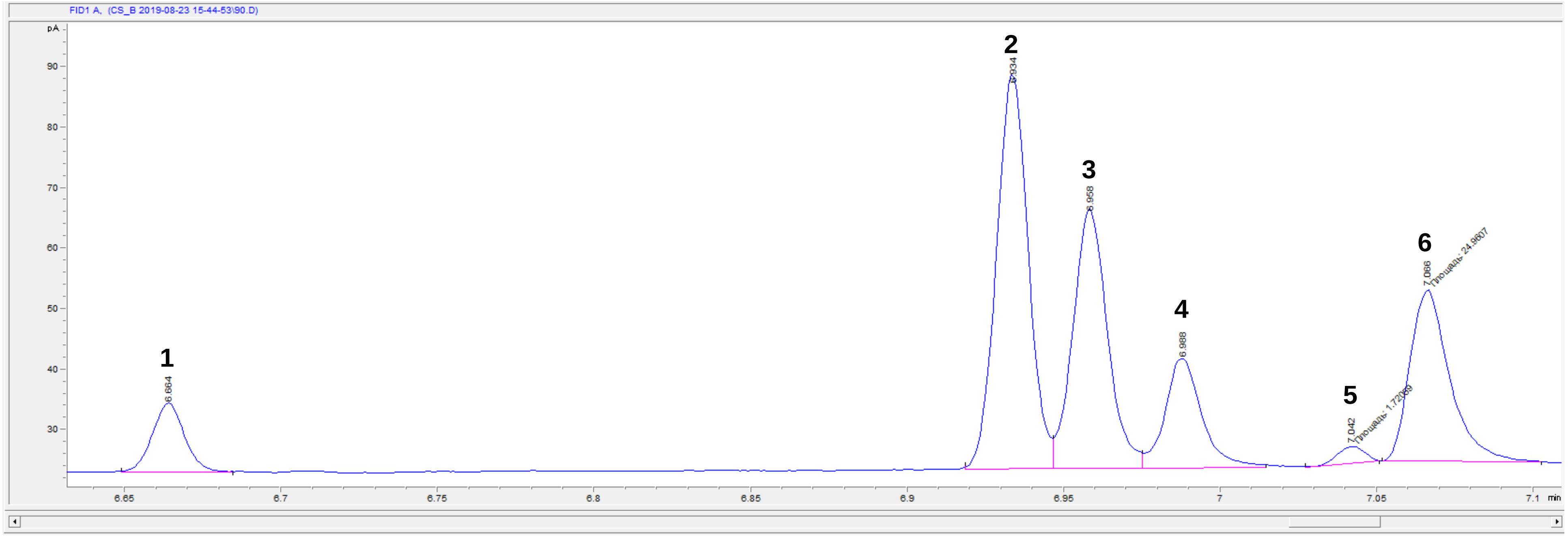

1 – *meso*-lactide, 2 – *l*-lactide, 3 – *l*-3-MG, 4 – *d*-3-MG, 5 – *d*-lactide, 6 – glycolide

Fig. S3. Chromatogram of depolymerization products of GAO:LAO with a ratio of 3:1 (Sample *A**_*γ*-Al_2O_3)

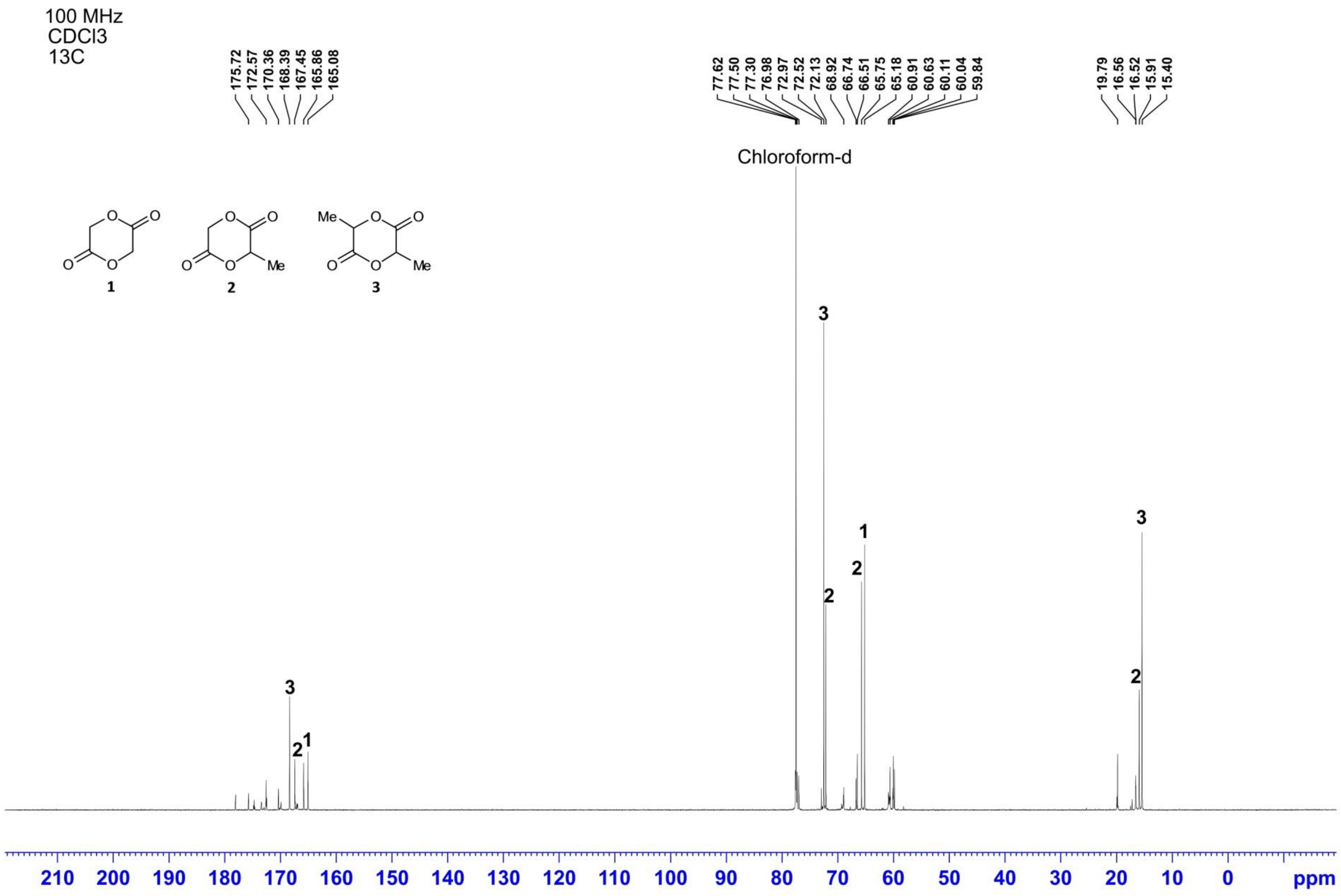

Fig. S4. ^{13}C NMR spectrum of depolymerization products of GAO:LAO with a ratio of 1:1 (Sample *B**_*γ-Al$_2$O$_3$*)

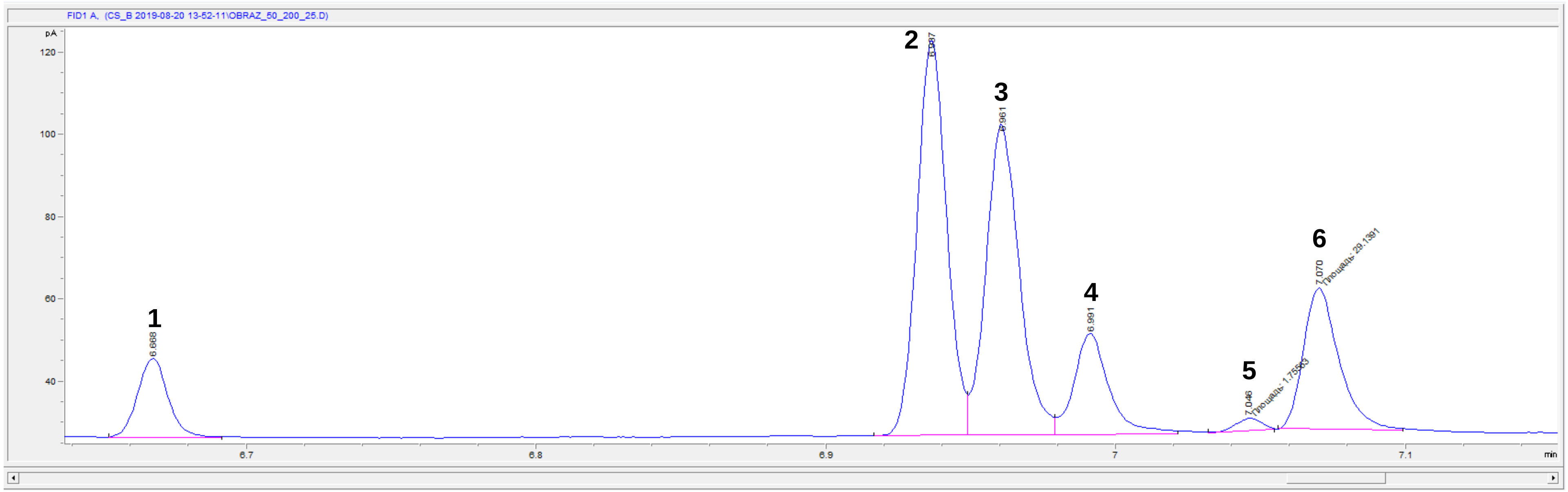

1 – *meso*-lactide, 2 – *l*-lactide, 3 – *l*-3-MG, 4 – *d*-3-MG, 5 – *d*-lactide, 6 – glycolide

Fig. S5. Chromatogram of depolymerization products of GAO:LAO with a ratio of 1:1 (Sample *B**_*γ*-Al_2O_3)

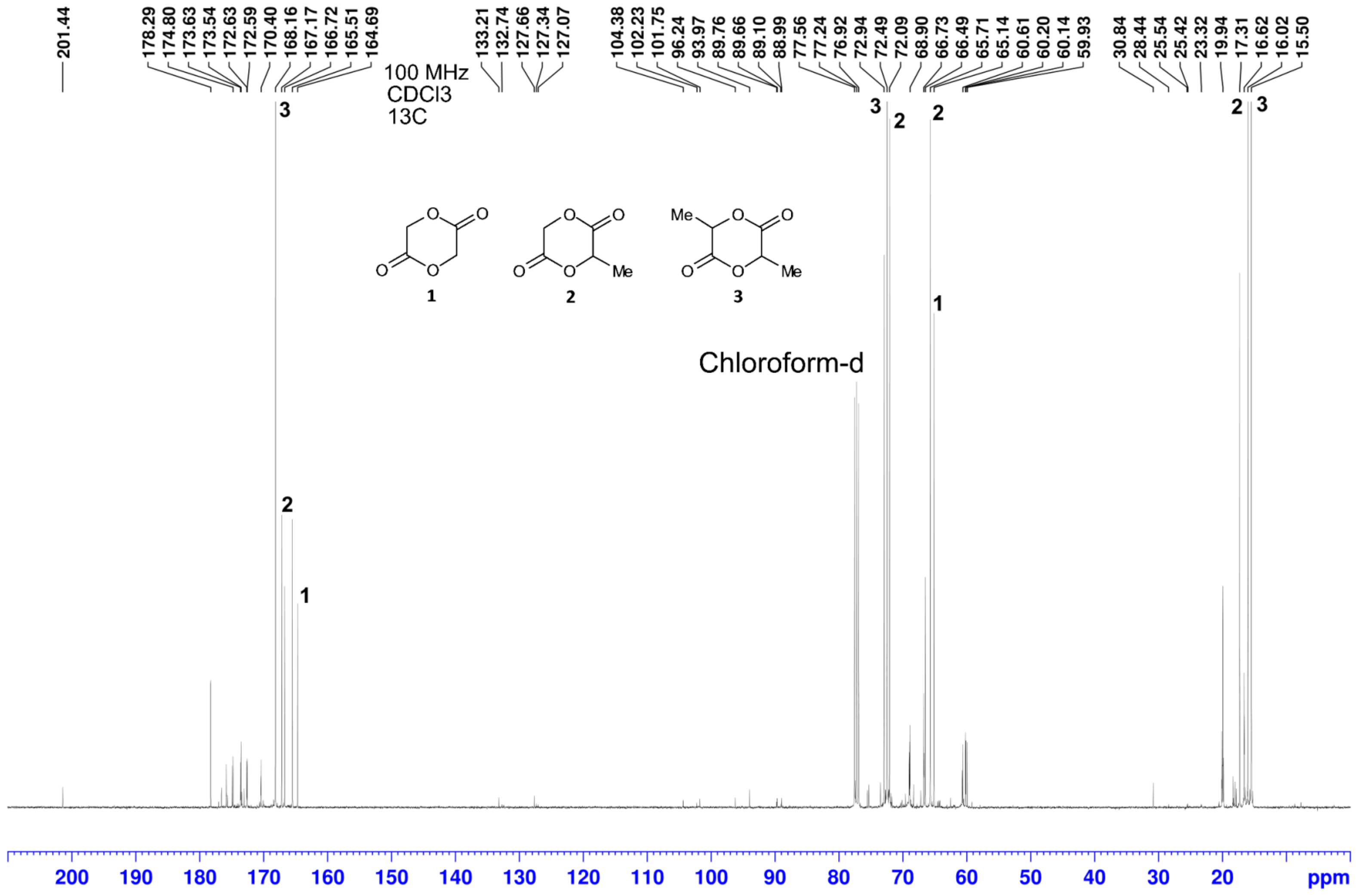

Fig. S6. ^{13}C NMR spectrum of depolymerization products of GAO:LAO with a ratio of 1:3 (Sample *C**_*γ-Al$_2$O$_3$*)

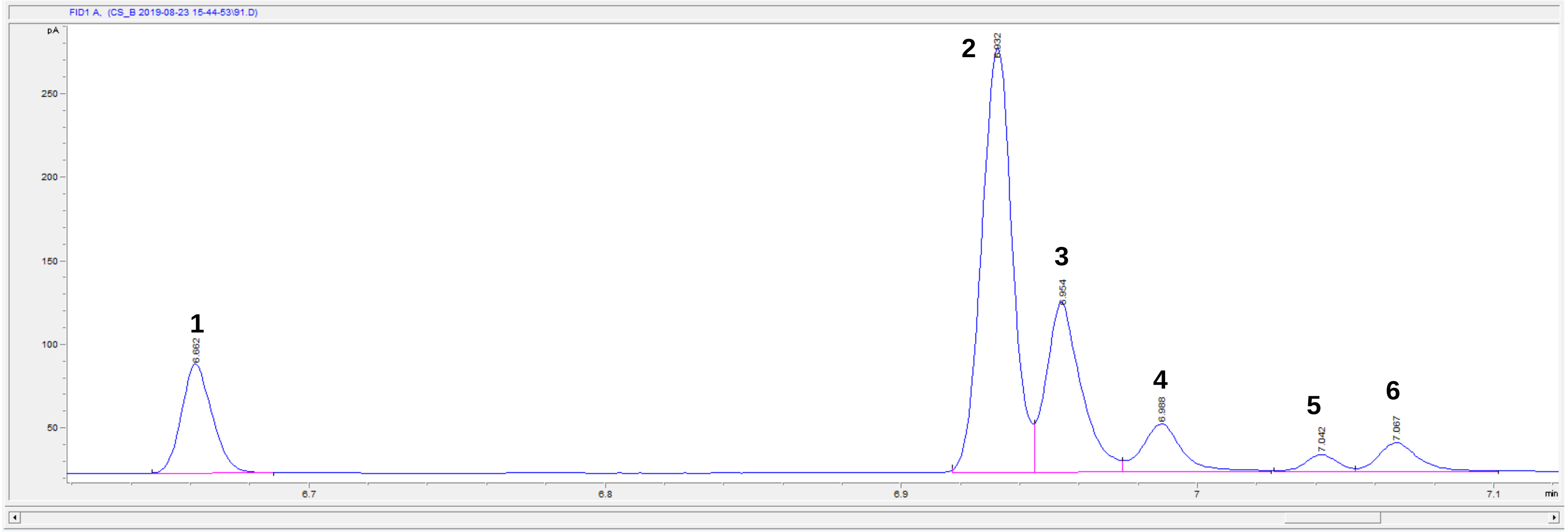

1 – *meso*-lactide, 2 – *l*-lactide, 3 – *l*-3-MG, 4 – *d*-3-MG, 5 – *d*-lactide, 6 – glycolide

Fig. S7. Chromatogram of depolymerization products of GAO:LAO with a ratio of 1:3 (Sample $C^*\_\gamma\text{-}Al_2O_3$)

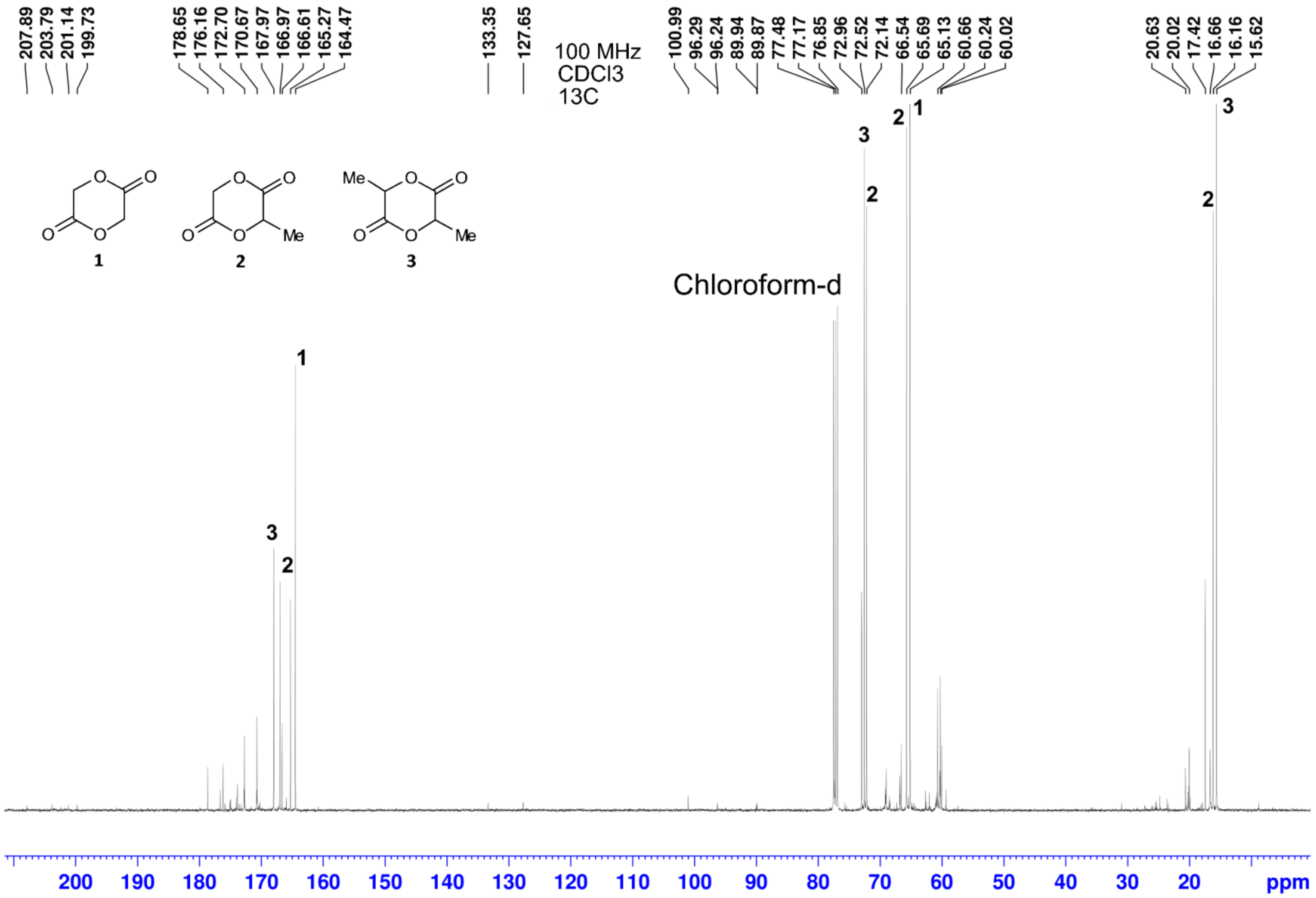

Fig. S8. ^{13}C NMR spectrum of depolymerization products of GAO:LAO with a ratio of 3:1 (Sample *D*_without catalyst*)

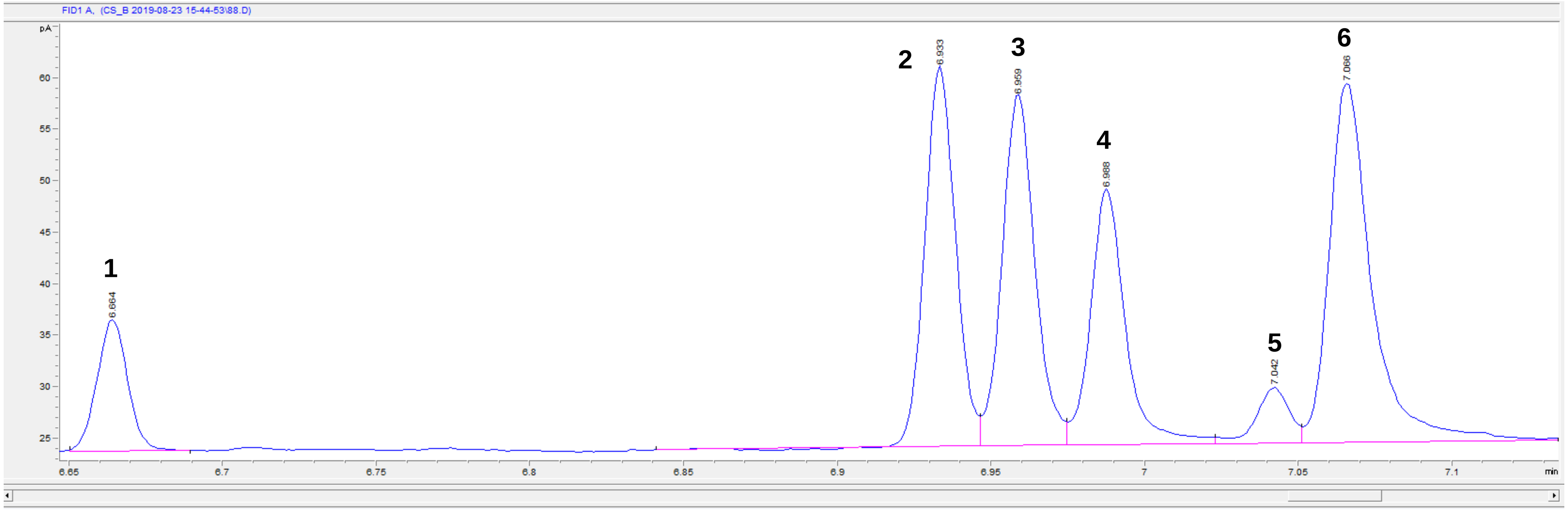

1 – *meso*-lactide, 2 – *l*-lactide, 3 – *l*-3-MG, 4 – *d*-3-MG, 5 – *d*-lactide, 6 – glycolide

Fig. S9. Chromatogram of depolymerization products of GAO:LAO with a ratio of 3:1 (Sample *D*_without catalyst*)

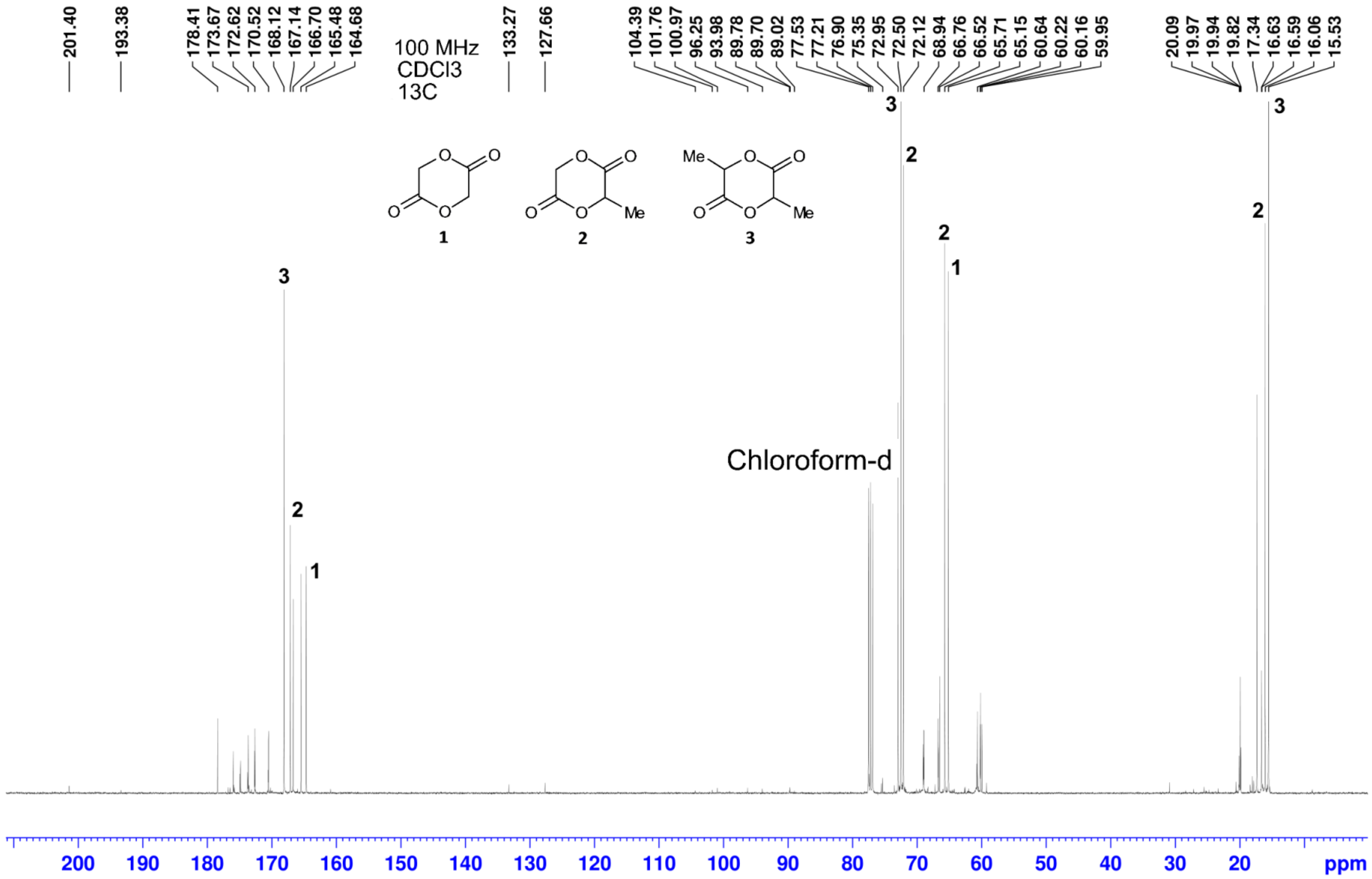

Fig. S10. ^{13}C NMR spectrum of depolymerization products of GAO:LAO with a ratio of 1:1 (Sample *E*_without catalyst*)

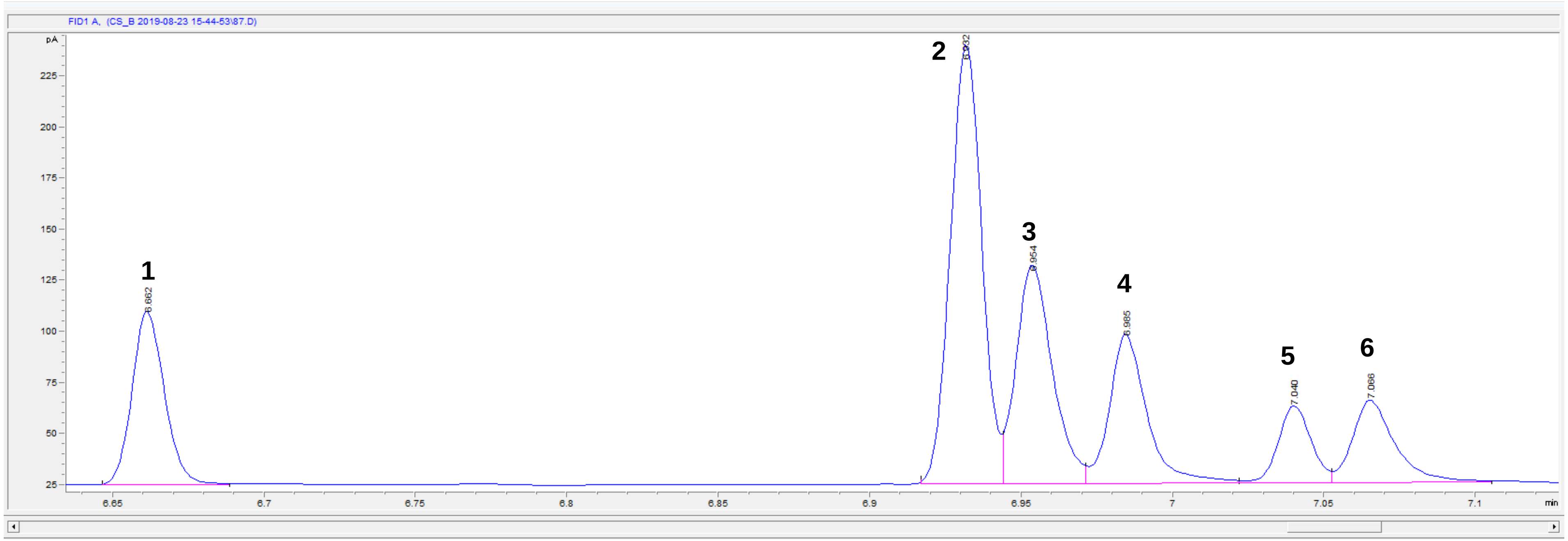

1 – *meso*-lactide, 2 – *l*-lactide, 3 – *l*-3-MG, 4 – *d*-3-MG, 5 – *d*-lactide, 6 – glycolide

Fig. S11. Chromatogram of depolymerization products of GAO:LAO with a ratio of 1:1 (Sample E*_*without catalyst*)

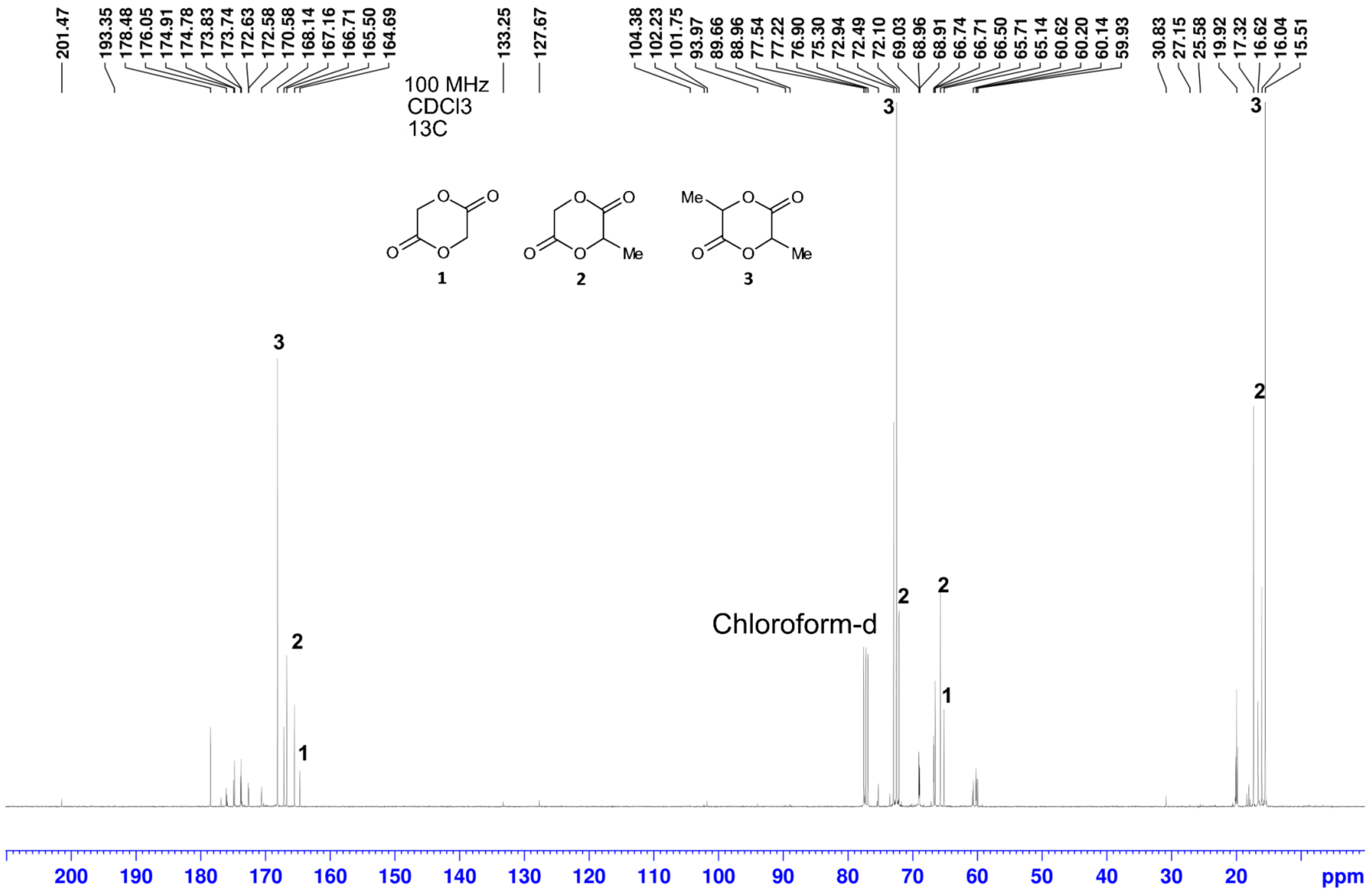

Fig. S12. ^{13}C NMR spectrum of depolymerization products of GAO:LAO with a ratio of 1:3 (Sample *F*_without catalyst*)

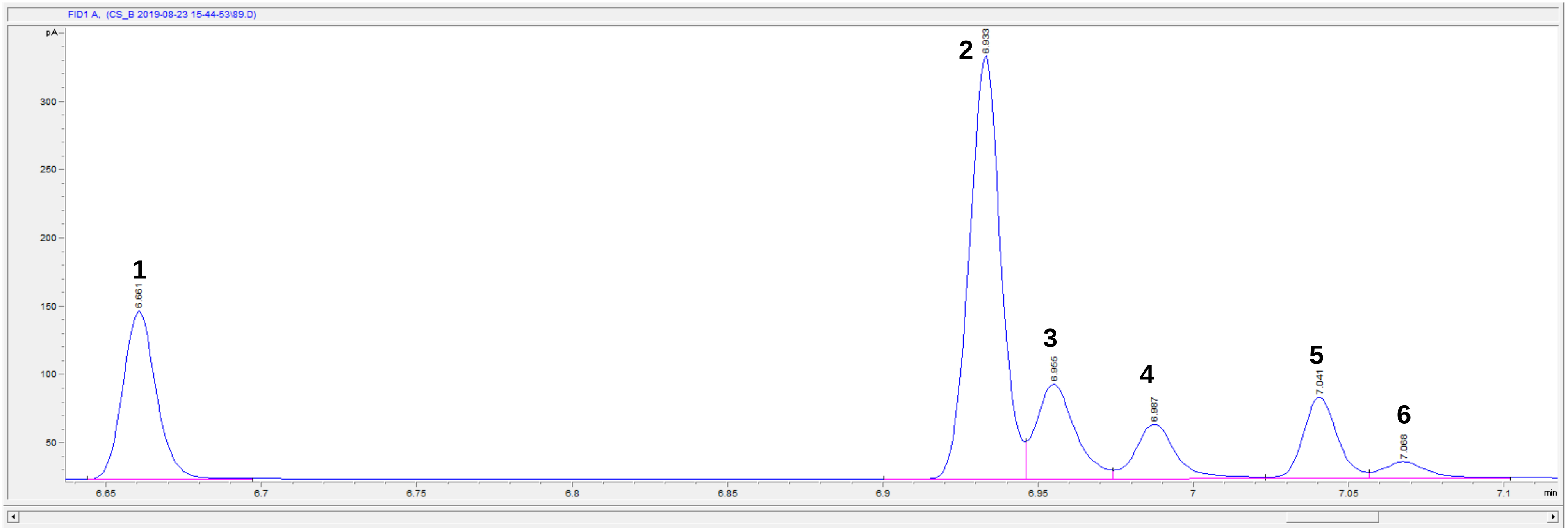

1 – *meso*-lactide, 2 – *l*-lactide, 3 – *l*-3-MG, 4 – *d*-3-MG, 5 – *d*-lactide, 6 – glycolide

Fig. S13. Chromatogram of depolymerization products of GAO:LAO with a ratio of 1:3 (Sample F*_*without catalyst*)

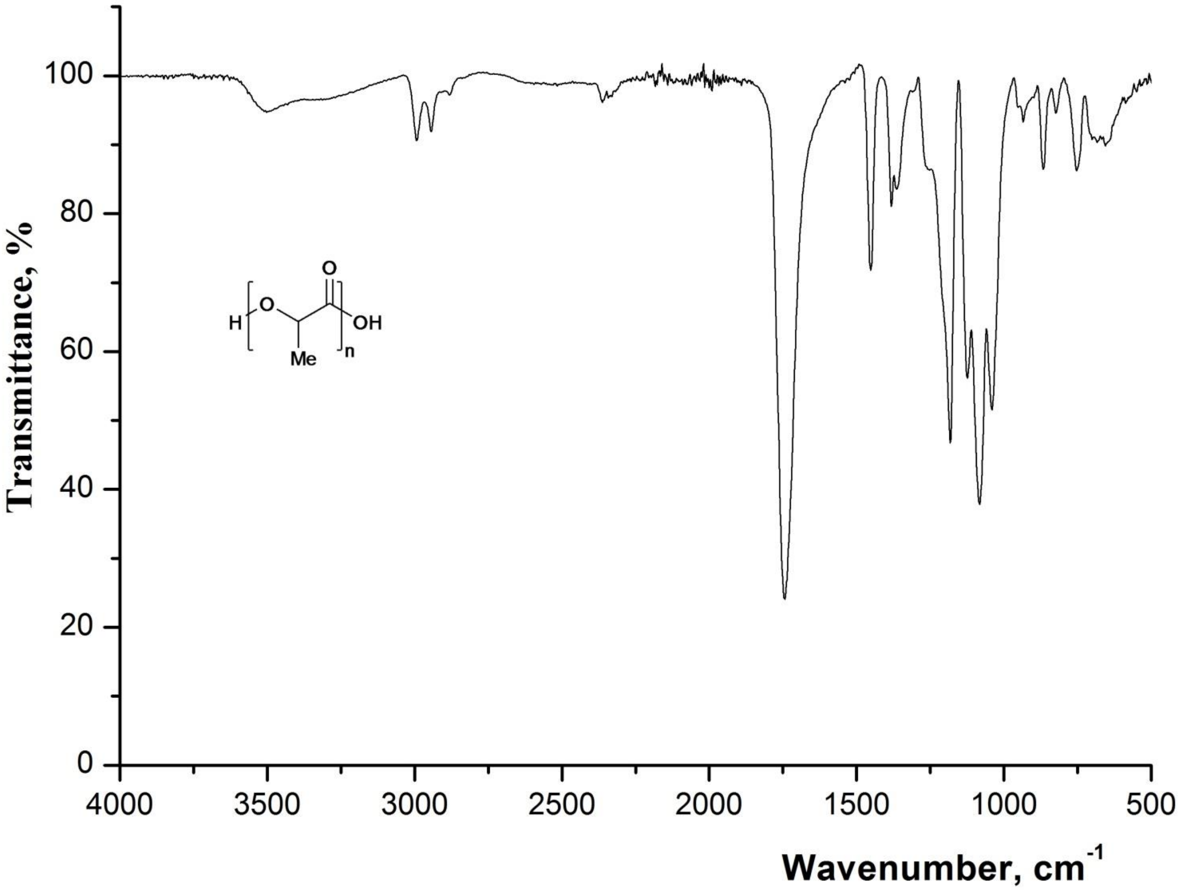

Fig. S14. IR spectrum of LAO

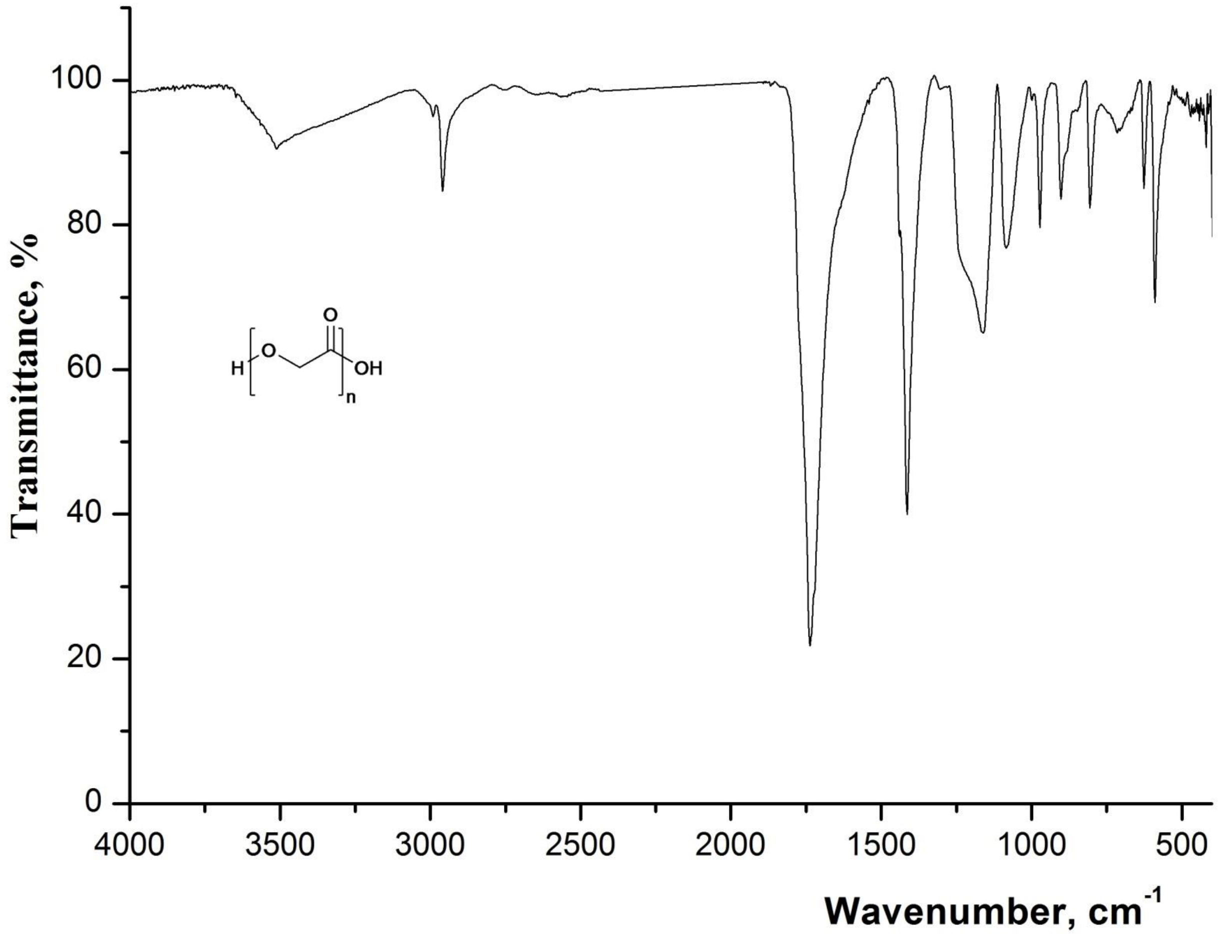

Fig. S15. IR spectrum of GAO

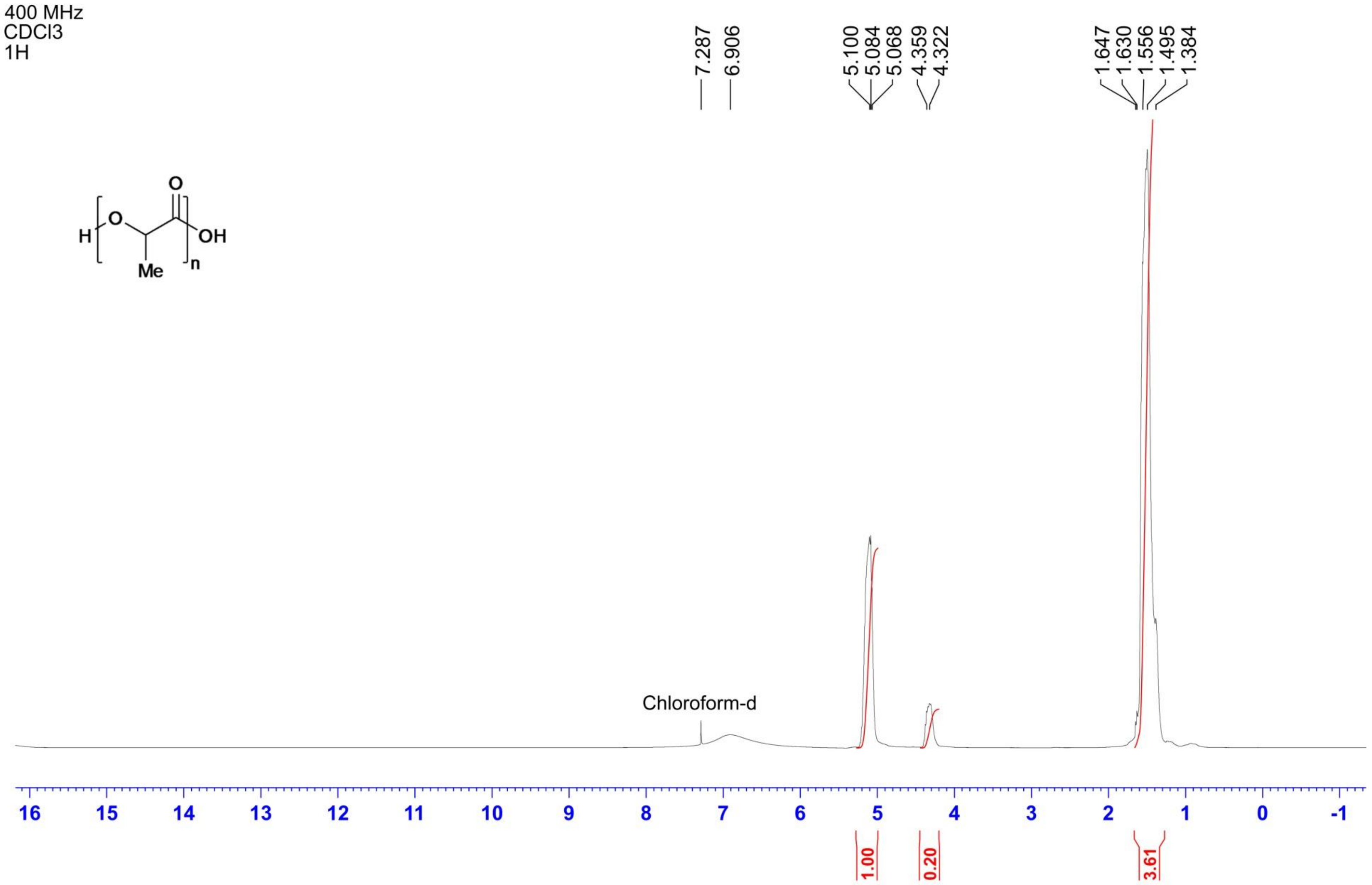

Fig. S16. NMR ^{1}H spectrum of LAO

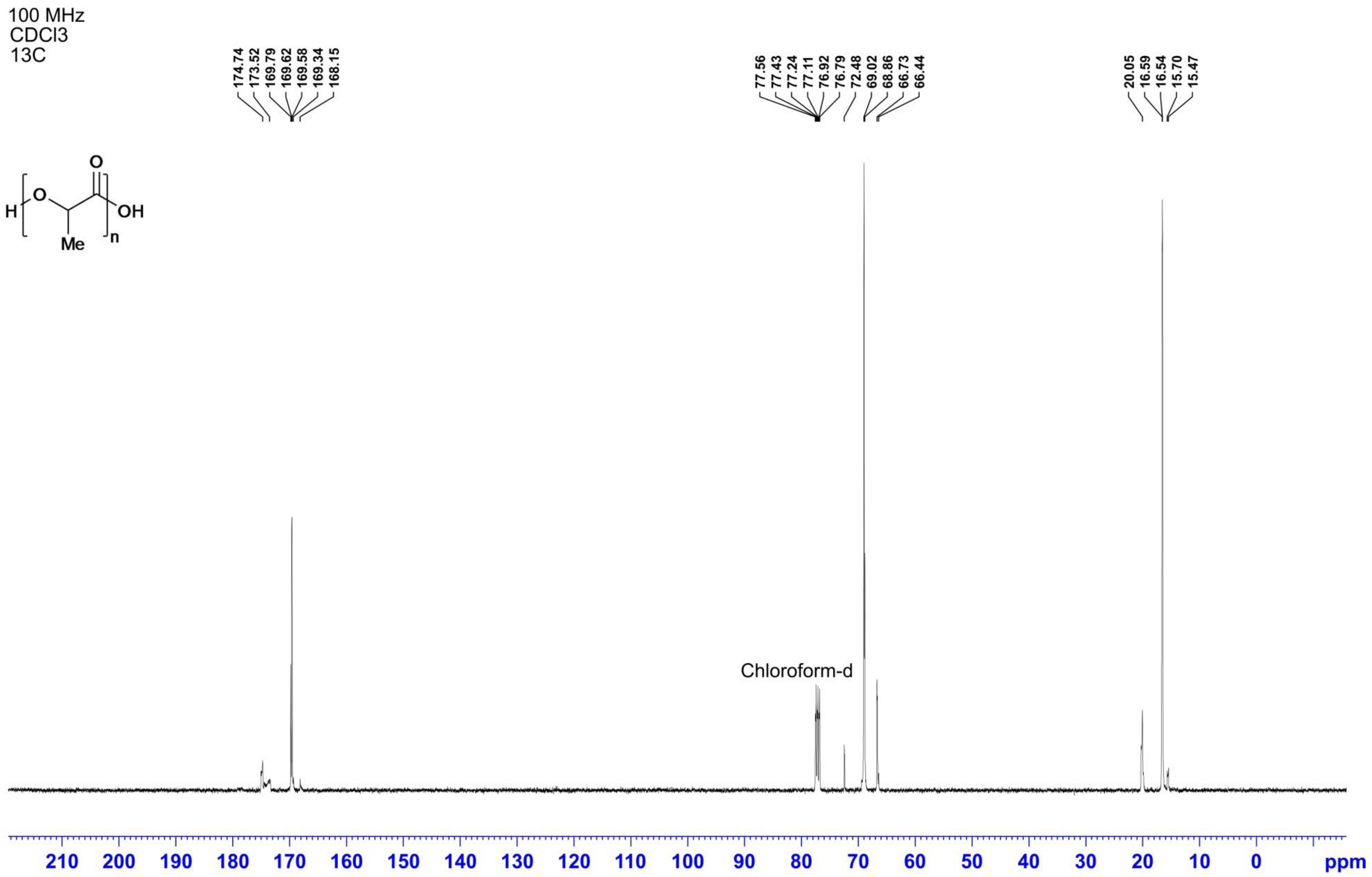

Fig. S17. NMR ^{13}C spectrum of LAO

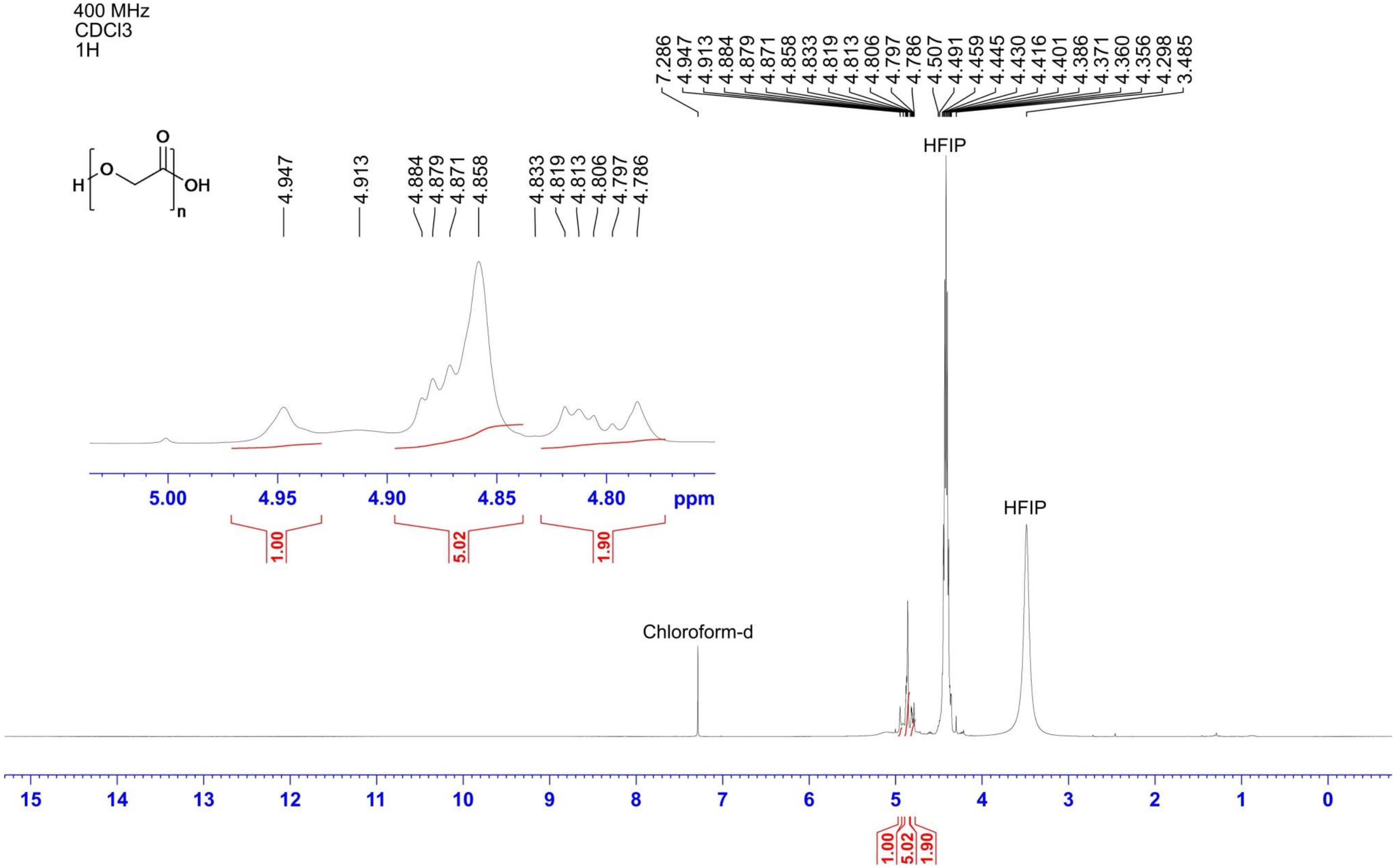

Fig. S18. NMR ^{1}H spectrum of GAO

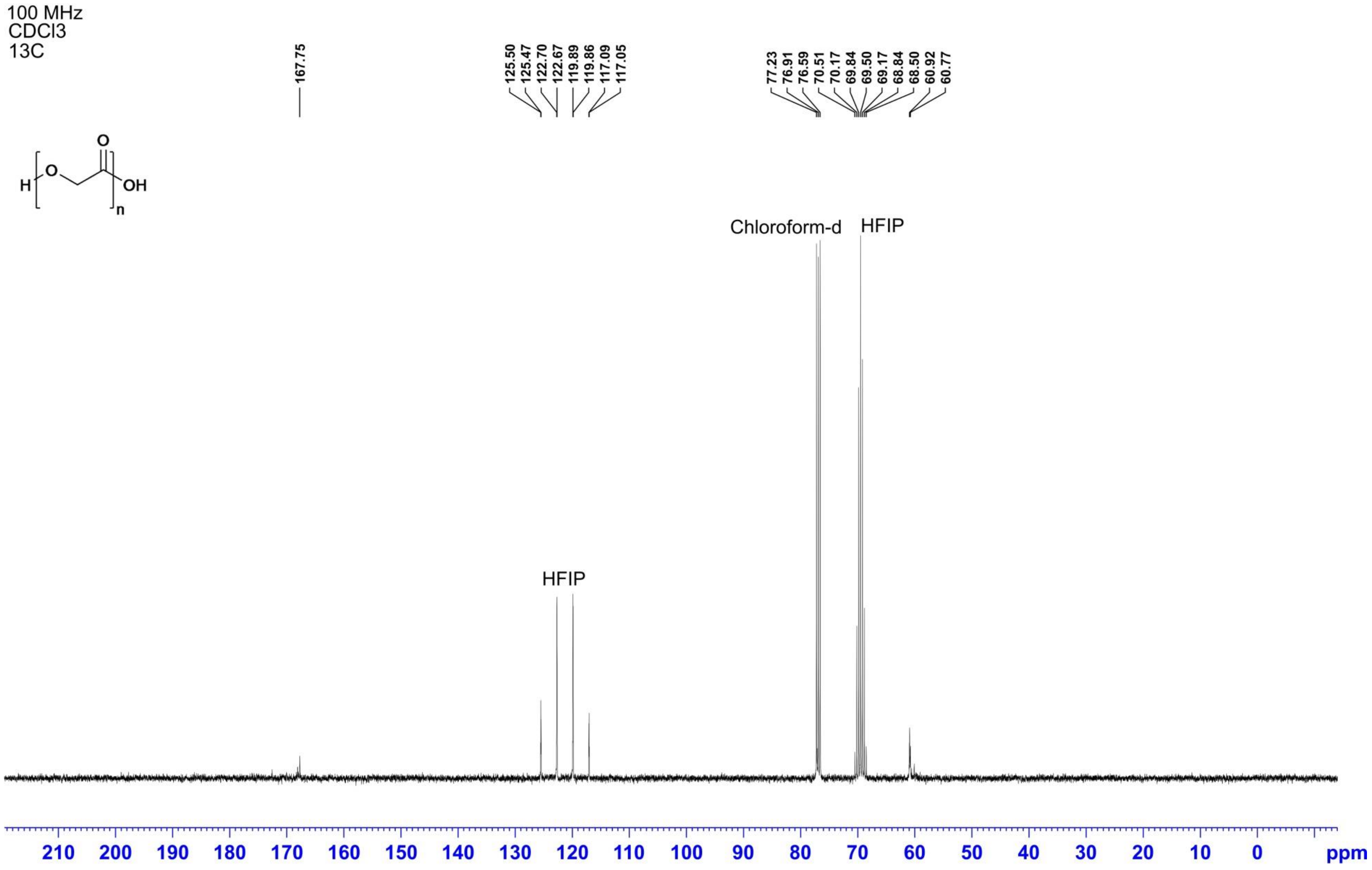

Fig. S19. NMR ^{13}C spectrum of GAO

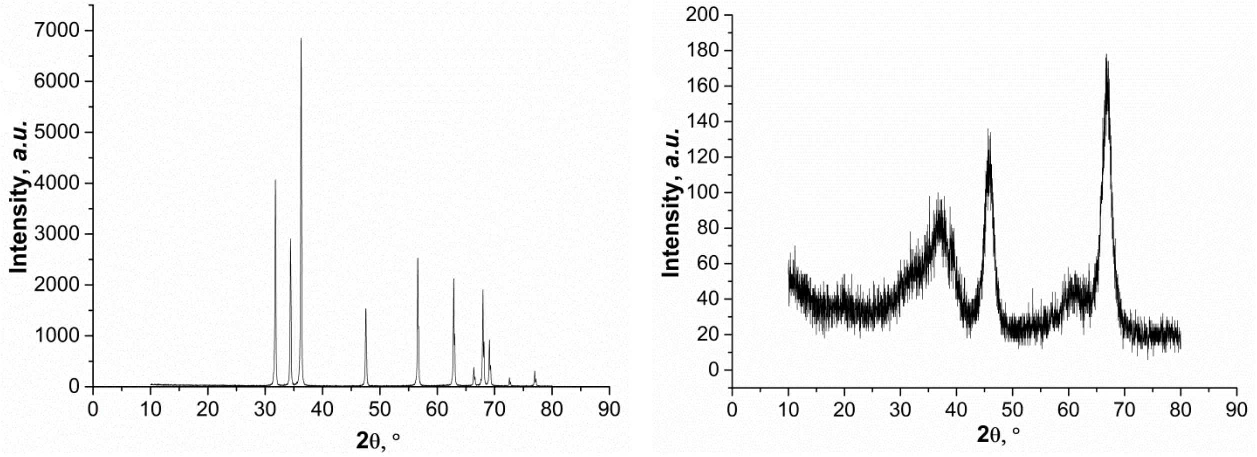

a *b*

a – ZnO, *b* – γ-Al_2O_3

Fig. S20. XRD patterns of depolymerization catalysts

Table S1. Phase composition of depolymerization catalysts

Catalyst	Phase (Space group)	Lattice parameters	CSR[a] (nm)
ZnO	Zincite ($P6_3mc$)	a = 3.2496 Å<br>c = 5.2063 Å	65
Al_2O_3	γ-Al_2O_3 ($Fd\bar{3}m$)	a = 7.8990 Å	5

[a] CSR = Coherent scattering region

Table S2. Surface properties of ZnO and γ-Al_2O_3

Catalyst	S_{BET}, m^2/g	BS[a] (type I)		BS (type II)		BS (type III)		ΣBS, μmol/g	$ΣBS/S_{BET}$, $μmol/m^2$	ΣLAS[d], μmol/g	$ΣLAS/S_{BET}$, $μmol/m^2$
		PA[b]	N[c]	PA	N	PA	N				
ZnO	3.4	970	6.5	–	–	870	4.5	11.0	3.24	10	2.94
γ-Al_2O_3	178.5	920	190.0	900	165	850	85.0	440.0	2.46	150	0.84

[a]BS=basic sites; [b]PA = proton affinity (in kJ/mol), [c]N = concentration of active sites (in μmol/g), [d]LAS = Lewis acid sites

ZnO: total pore volume = 0.015 cm^3/g, average pore size = 17.82 nm

γ-Al_2O_3: total pore volume = 0.416 cm^3/g, average pore size = 9.33 nm

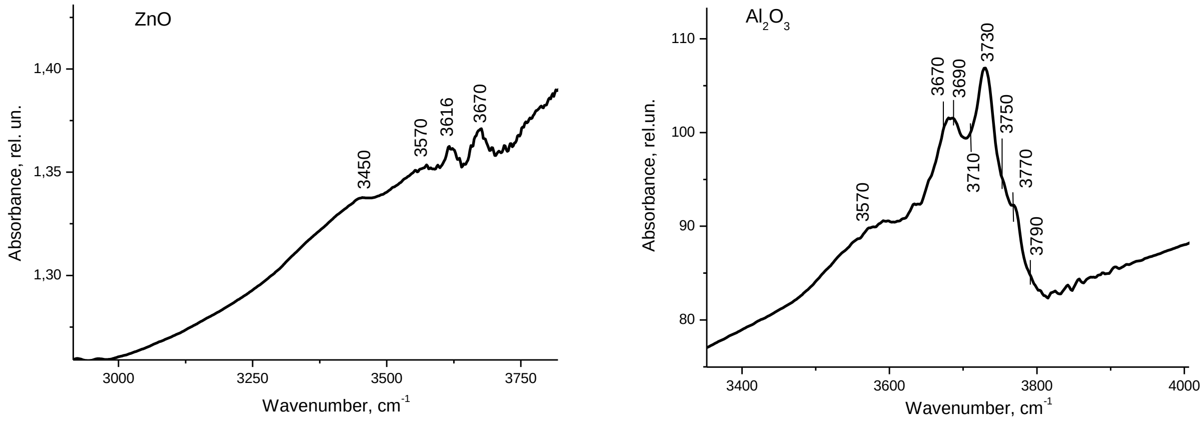

a *b*

Fig. S21. IR spectra of oxides at the region of hydroxyl groups: ZnO (*a*), γ-Al_2O_3 (*b*)

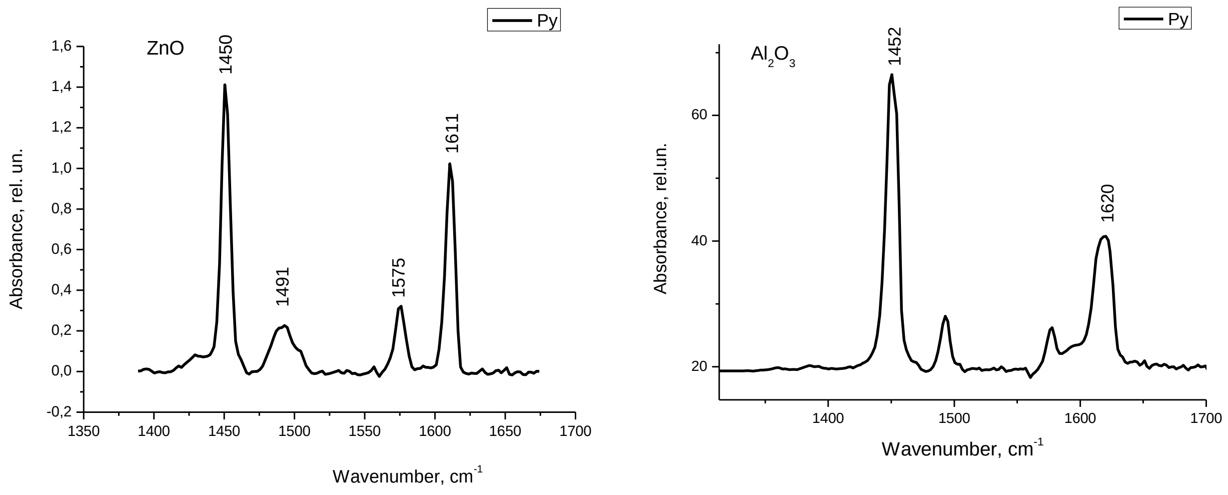

a *b*

Fig. S22. IR spectra of adsorbed pyridine on the surface of ZnO (*a*) and γ-Al_2O_3 (*b*)

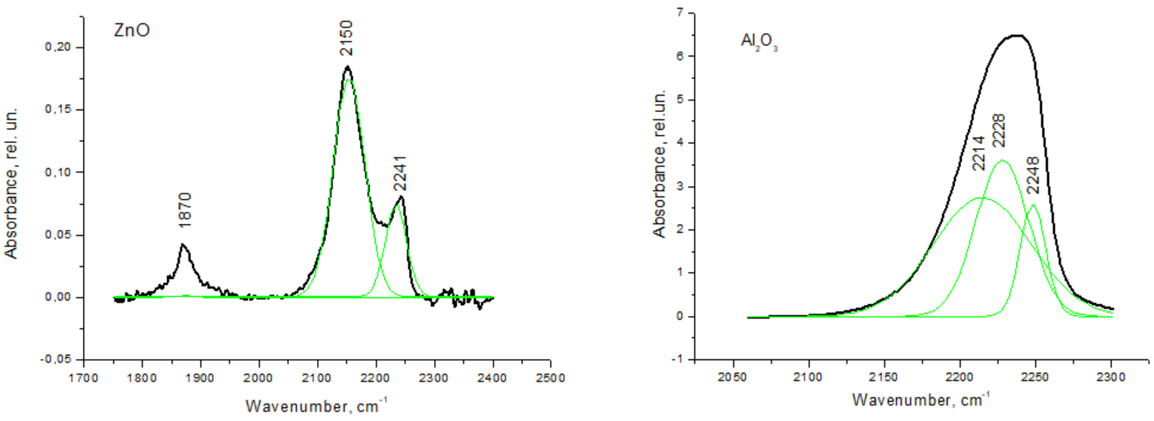

a *b*

Fig. S23. IR spectra of adsorbed $CDCl_3$ on the surface of ZnO (*a*) and γ-Al_2O_3 (*b*)

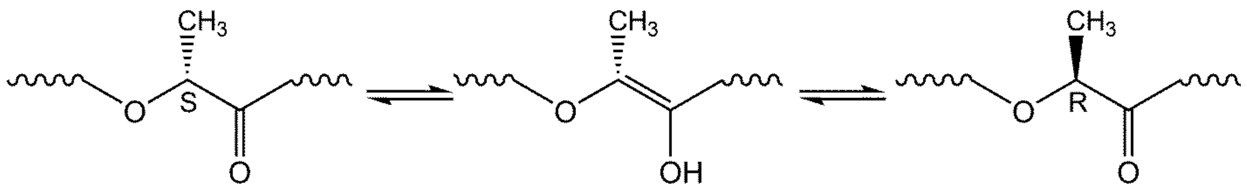

Fig. S24. Possible route of LAO epimerization

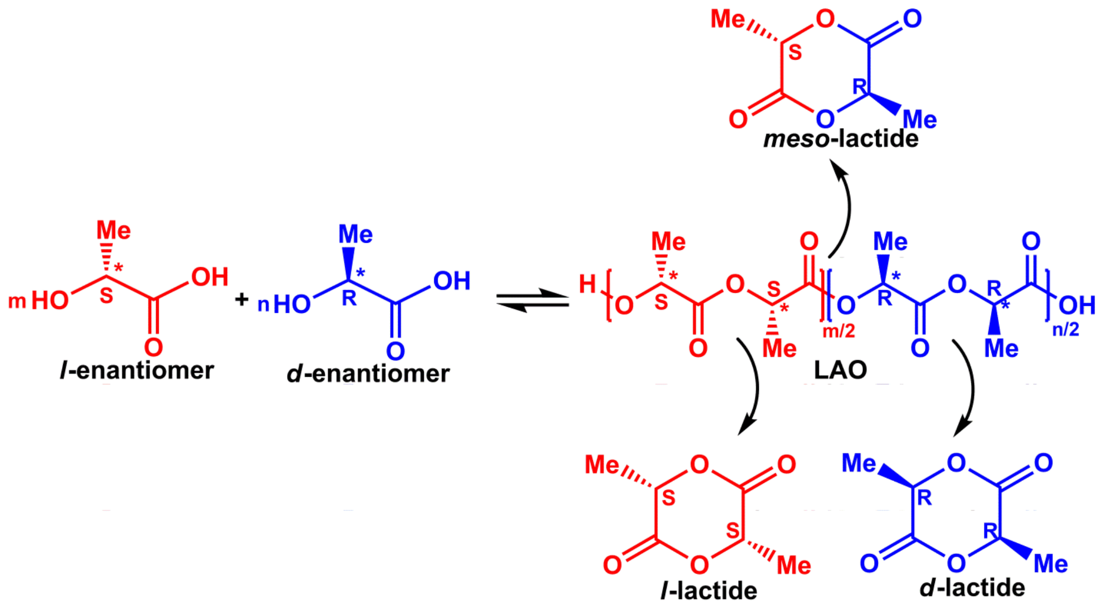

Fig. S25. Routes of *meso*- and *d*-lactide formation

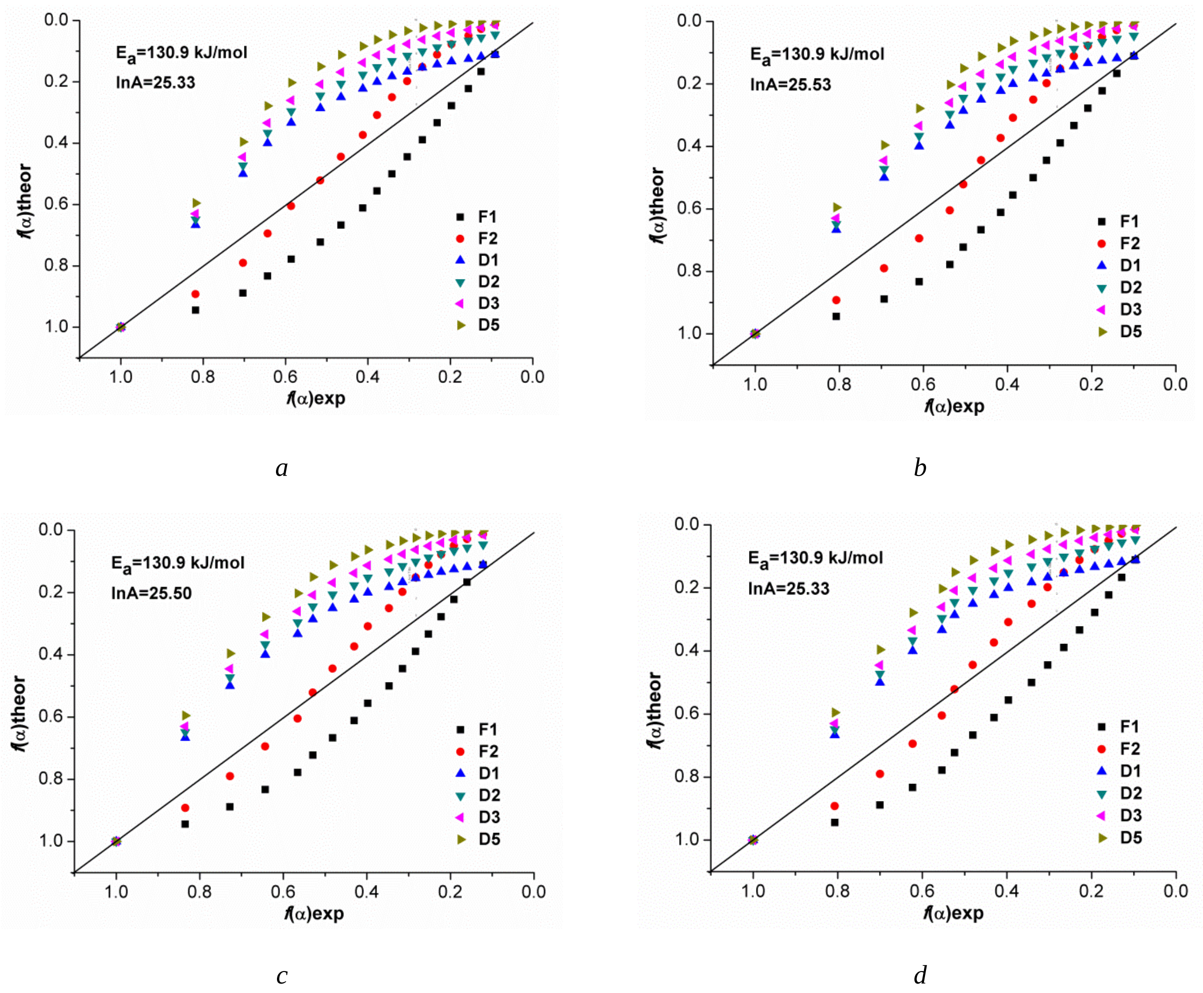

a – 5 K/min, *b* – 10 K/min, *c* – 15 K/min, *d* – 20 K/min

Fig.S26. Comparison of experimental and theoretical *f*(α) for depolymerization in the presence of ZnO

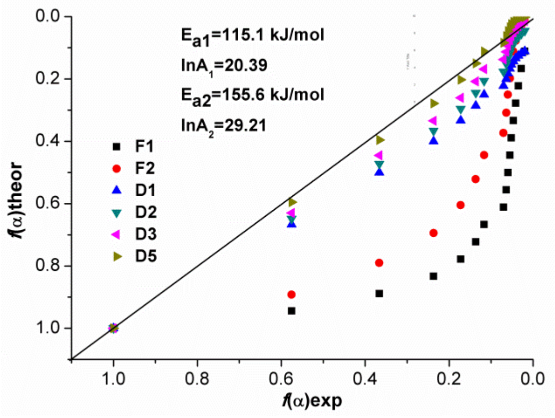

a

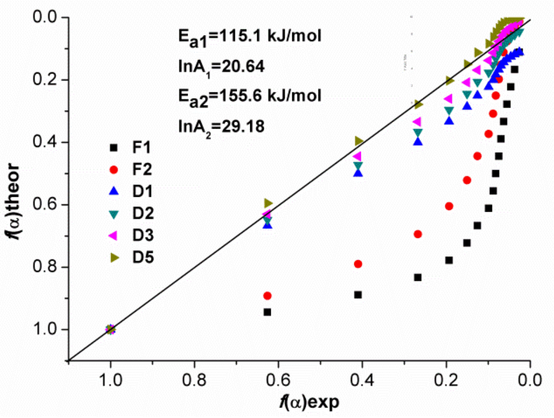

b

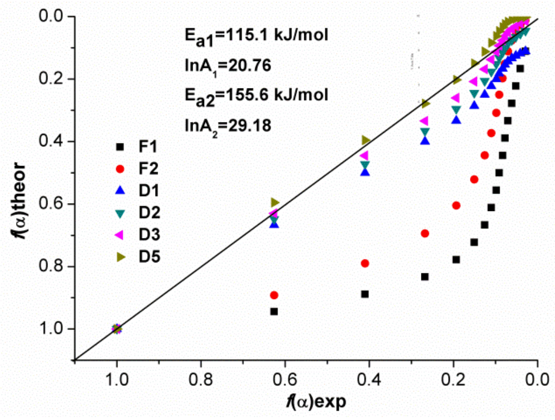

c

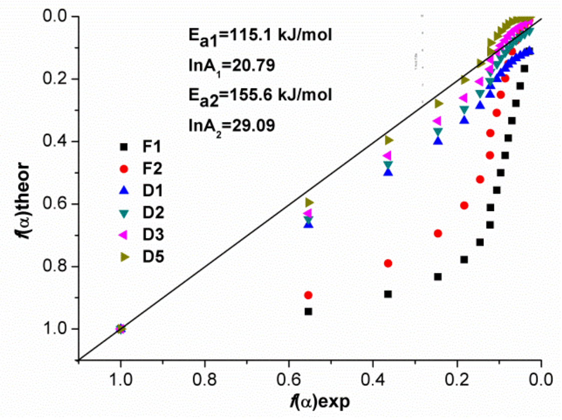

d

a – 5 K/min, *b* – 10 K/min, *c* – 15 K/min, *d* – 20 K/min

Fig.S27. Comparison of experimental and theoretical *f*(α) for depolymerization in the presence of γ-Al_2O_3

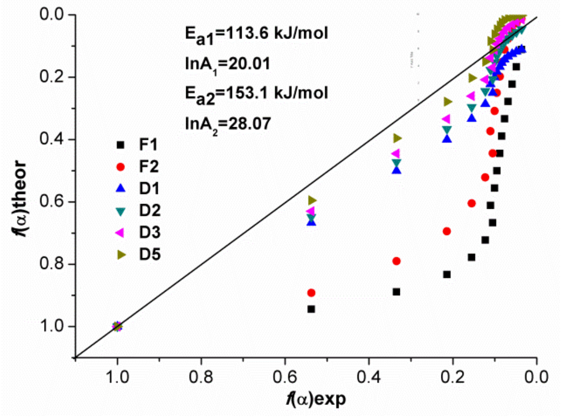

a

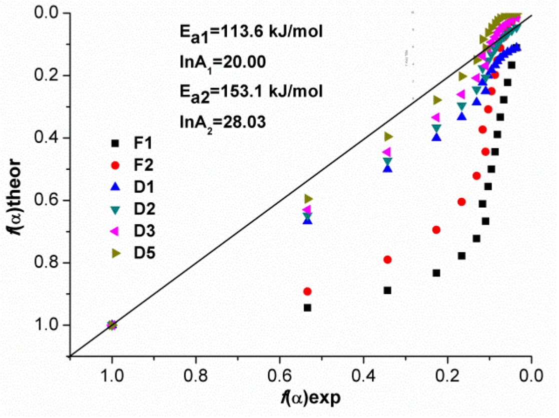

b

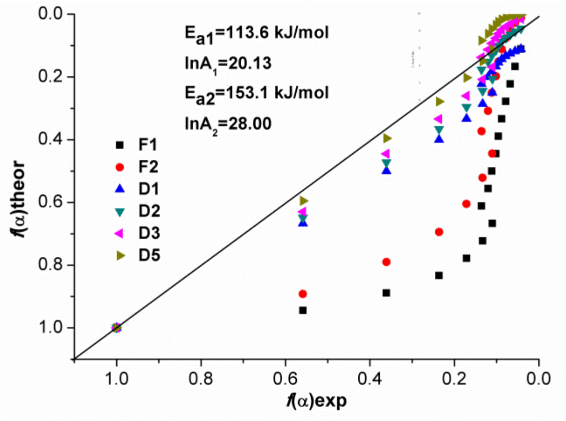

c

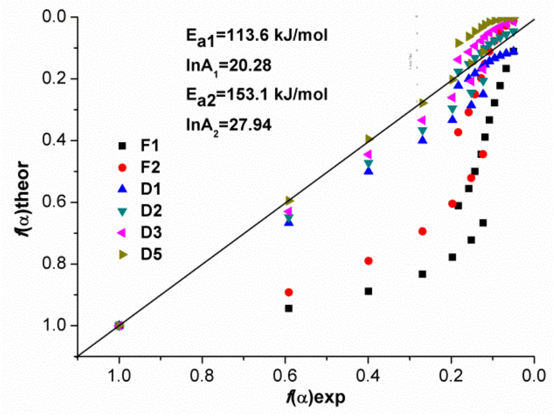

d

a – 5 K/min, *b* – 10 K/min, *c* – 15 K/min, *d* – 20 K/min

Fig.S28. Comparison of experimental and theoretical *f*(α) for depolymerization without catalyst
